# New Insights on Mt. Etna’s Crust and Relationship with the Regional Tectonic Framework from Joint Active and Passive P-Wave Seismic Tomography

**DOI:** 10.1007/s10712-017-9425-3

**Published:** 2017-09-15

**Authors:** A. Díaz-Moreno, G. Barberi, O. Cocina, I. Koulakov, L. Scarfì, L. Zuccarello, J. Prudencio, A. García-Yeguas, I. Álvarez, L. García, J. M. Ibáñez

**Affiliations:** 1 Department of Earth, Ocean and Ecological Sciences, School of Environmental Sciences, Jane Herdman Building, 4 Brownlow Street, Liverpool, Merseyside L69 3GP UK; 2 Instituto Andaluz de Geofisica, University of Granada, 18071 Granada, Spain; 3 Istituto Nazionale di Geofisica e Vulcanologia, Sezione di Catania - Osservatorio Etneo, 95125 Catania, Italy; 4 Trofimuk Institute of Petroleum Geology and Geophysics SB RAS, Prospekt Koptyuga, 3, Novosibirsk, Russia 630090; 5 Novosibirsk State University, Pirogova str., 2, Novosibirsk, Russia 630090; 6 Department of Applied Physics, University of Cadiz, 11510 Cádiz, Spain; 7 Department of Communication and Signal Theory, University of Granada, 18071 Granada, Spain; 8 Department of Earth and Planetary Science, University of California at Berkeley, Berkeley, CA 94709 USA; 9 Department of Theoretical Physics and Cosmos, University of Granada, 18071 Granada, Spain

**Keywords:** Seismic tomography, Mt. Etna, Aeolian Islands, Volcanic structure

## Abstract

In the Central Mediterranean region, the production of chemically diverse volcanic products (e.g., those from Mt. Etna and the Aeolian Islands archipelago) testifies to the complexity of the tectonic and geodynamic setting. Despite the large number of studies that have focused on this area, the relationships among volcanism, tectonics, magma ascent, and geodynamic processes remain poorly understood. We present a tomographic inversion of P-wave velocity using active and passive sources. Seismic signals were recorded using both temporary on-land and ocean bottom seismometers and data from a permanent local seismic network consisting of 267 seismic stations. Active seismic signals were generated using air gun shots mounted on the Spanish Oceanographic Vessel ‘Sarmiento de Gamboa’. Passive seismic sources were obtained from 452 local earthquakes recorded over a 4-month period. In total, 184,797 active P-phase and 11,802 passive P-phase first arrivals were inverted to provide three different velocity models. Our results include the first crustal seismic active tomography for the northern Sicily area, including the Peloritan–southern Calabria region and both the Mt. Etna and Aeolian volcanic environments. The tomographic images provide a detailed and complete regional seismotectonic framework and highlight a spatially heterogeneous tectonic regime, which is consistent with and extends the findings of previous models. One of our most significant results was a tomographic map extending to 14 km depth showing a discontinuity striking roughly NW–SE, extending from the Gulf of Patti to the Ionian Sea, south-east of Capo Taormina, corresponding to the Aeolian–Tindari–Letojanni fault system, a regional deformation belt. Moreover, for the first time, we observed a high-velocity anomaly located in the south-eastern sector of the Mt. Etna region, offshore of the Timpe area, which is compatible with the plumbing system of an ancient shield volcano located offshore of Mt. Etna.

## Introduction

The Mediterranean represents a complex tectonic region located between the African and Eurasian plates. In this area, large-scale extensional basins and convergent domains coexist with a diverse range of volcanic environments. Given this complexity, the Mediterranean basin cannot be explained using a uniform geodynamic framework. The Western–Central Mediterranean is dominated by extensional basins in a back-arc setting, including the Alboran Sea to the west, and the Thyrrenian and Ionian basins in the central area (e.g., Monna et al. 2013; Argnani et al. 2016). The Eastern Mediterranean (Aegean region) has historically been considered an ancient back-arc extensional basin associated with the north-east Hellenic arc subduction zone, which at present is characterized by little or no tectonic activity (e.g., McClusky et al. 2000; Papanikolaou and Royden 2007). Over the past two decades, numerous approaches have been used to describe, interpret, and model the geodynamics of the Mediterranean region. In particular, in the southern Tyrrhenian Sea and eastern Sicily, several studies have attempted to constrain the structure of the crust (e.g., Barberi et al. 2004; Scarfì et al. 2007, 2016).

**Fig. 1:**
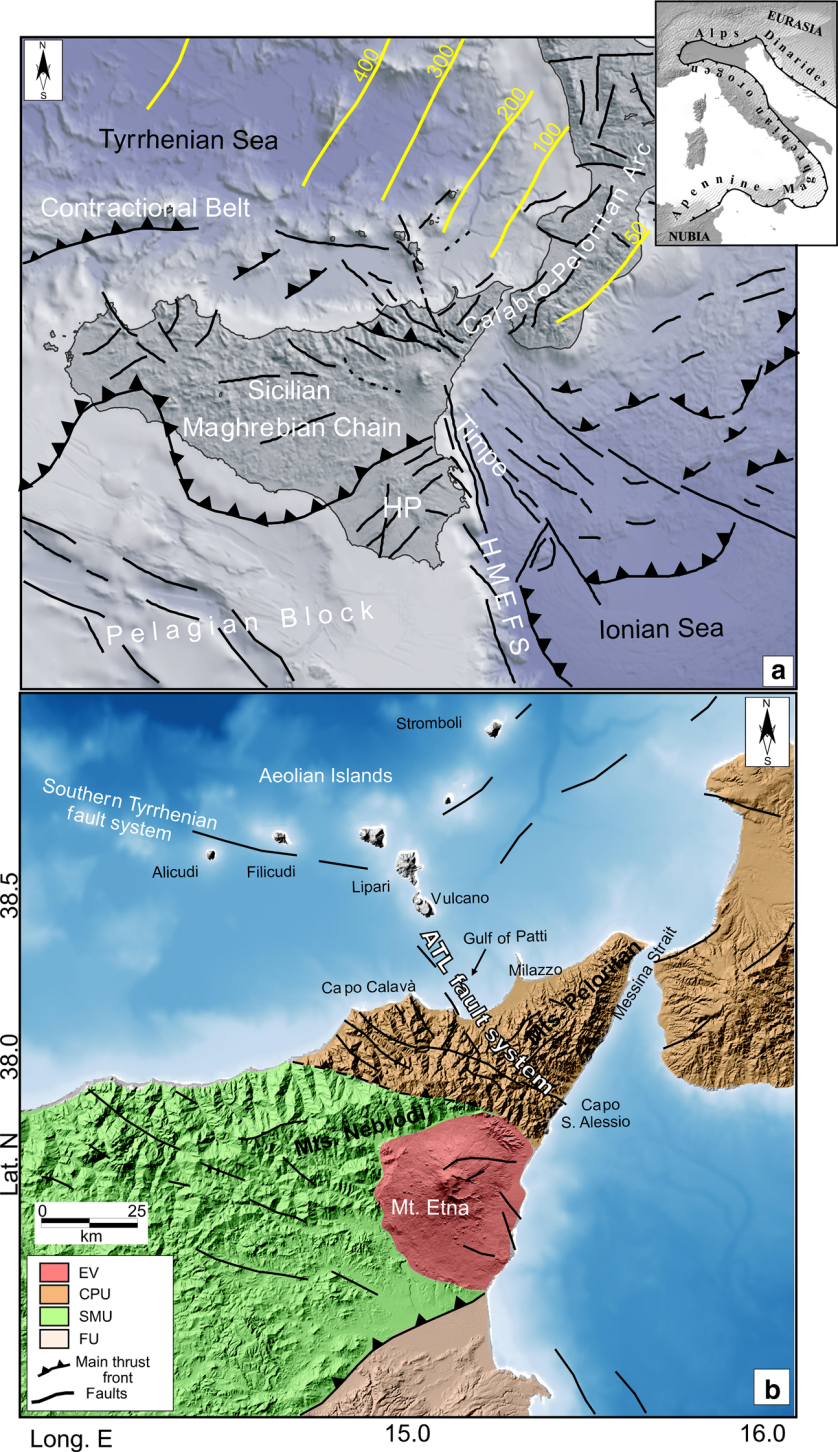
**a** Structural setting of the Mediterranean Basin showing the main contact regions between the European and African plates and the position of the main active volcanoes. **b**, **c** Simplified tectonic and geological maps (after Barreca et al. 2014; Musumeci et al. 2014; Scarfì et al. 2016). Abbreviations are as follows: HP, Hyblean Plateau; HMEFS, Hyblean-Maltese Escarpment Fault System; ATL, Aeolian–Tindari–Letojanni fault system; EV, Etnean volcanics; CPU, Calabro-Peloritan units; and SMU, Sicilian–Maghrebian units. Yellow lines in Fig. 1b represent the isodepths of the subducting Ionian slab (from Selvaggi and Chiarabba 1995)

The geodynamic complexity of the Mediterranean region is also reflected in the variety of volcanic products and eruption styles, including: (a) an absence of active volcanism in the westernmost margin (Alboran Sea); (b) very active Italian volcanoes such as Mt. Etna, Stromboli, Vesuvius, and Campi Flegrei; and (c) varied and complex Greek Islands such as Nisyros and Santorini (Fig. 1). The role of volcanic activity in the complex regional geodynamic setting has been noted by several studies (e.g., Barberi et al. 1973; Beccaluva et al. 1985; Doglioni et al. 1994, 2001; Mantovani et al. 1996; Branca et al. 2008; Chiarabba et al. 2008). Among the volcanic centres, Nisyros Island is composed of basaltic–andesitic–rhyolitic products representing both early-stage large-scale basaltic flows and Plinian eruptive episodes that formed the present-day caldera (e.g., Caliro et al. 2005 and references therein). The Santorini volcanic complex is characterized by periods of basaltic shield volcanism interrupted by large explosive events (e.g., Dimitriadis et al. 2005 and references therein). Mt. Vesuvius has produced a variety of magma compositions, from basaltic to andesitic, and is characterized by Plinian and sub-Plinian episodes followed by a long period of Strombolian and effusive activity (e.g., Saccorotti et al. 2002 and references therein). Activity in Sicily and the Southern Tyrrhenian is a mixture of intraplate and arc volcanism that overlapped in space and time (e.g., Rosenbaum and Lister 2004; Faccenna et al. 2005). Furthermore, in addition to physical and chemical differences, the ages of volcanic activity range significantly, for example, 7.0–1.1 Ma on the Hyblean Plateau (Fig. 1), 0.5 Ma to present at Mt. Etna, and 1.3 Ma to present in the Aeolian Islands archipelago.

In addition to volcanic activity, eastern Sicily and the Calabrian Arc, together with the Apennine chain, also represent one of the most active tectonic zones in the Central Mediterranean region, as demonstrated by the high number of significant earthquakes that have occurred in recorded history (e.g., the 1169 and 1693 earthquakes, the sequence of 1783 earthquakes in southern Calabria, and the 1908 earthquake in the Strait of Messina).

Improved understanding of the structure and dynamics the volcanic environment is needed to better understand the relationship between volcanism and its regional framework. Identifying the interaction between volcanism and tectonics and the characteristics of volcano-dynamic behaviour are necessary for understanding the interplay between tectonics, deformation processes, and magma transport through the lithosphere (e.g., Vigneresse 1999; Petford et al. 2000); therefore, both the geophysical characteristics of a volcano and its evolution play a major role in reconstructing regional geodynamic models.

The mechanisms involved in sub-surface magma migration and dynamics remain poorly constrained, mainly owing to the absence of direct information (Jaxybulatov et al. 2014). To obtain the relevant data, indirect geophysical methods are needed, including magnetotelluric studies (Hill et al. 2009), gravimetric analysis (Carbone et al. 2013, 2014; Cannavò et al. 2015), and/or seismic tomography (e.g., Lees 2007; Zandomeneghi et al. 2009; García-Yeguas et al. 2014). In particular, seismic tomography has been shown to be one of the most powerful and effective approaches for investigating sub-surface volcanic features down to significant depths (Koulakov and Shapiro 2015).

In this study, we aimed to better constrain the volcanic regions associated with Mt. Etna and the Aeolian Islands archipelago within a regional tectonic framework using a joint active and passive P-wave seismic tomographic inversion. We provided new tomographic results for the crust beneath north-eastern Sicily, including Mt. Etna and the Aeolian Islands archipelago using data acquired during the TOMO-ETNA experiment (Ibáñez et al. 2016a, b; Coltelli et al. 2016) performed between June and November 2014 under the EC-FP7 MED-SUV and EUROFLEET2 MED-SUV.ISES projects. The main goal of the TOMO-ETNA experiment was to better define the crustal structures and the main regional fault systems and inform on the physical processes controlling magma ascent beneath Mt. Etna and the Aeolian Island volcanoes. TOMO-ETNA, which was the most complete and complex seismic survey ever performed in the region, combined active and passive seismic sources with a dense seismic network that included both on-land and ocean bottom seismometers (OBS). In the present work, we considered a region extending from the Aeolian Islands to the Mt. Etna volcanic edifice and its offshore environs. Furthermore, we performed two additional inversions to enhance the resolution of the tomographic models beneath both volcanic regions.

## Tectonic Setting

Sicily is located in the Central Mediterranean (Fig. 1a, b). It extends across the Calabrian accretionary wedge, linking the Apennines mountain belt with the African Maghrebides (Fig. 1b). The island shows evidence for a transition between extensional and convergent crustal zones and the observed tectonic structures play an important role in both volcanism and seismicity of the Central Mediterranean. In particular, Mt. Etna (eastern Sicily) and the Aeolian volcanic arc represent two of the most important regional geological features.

These volcanic complexes are located between different structural domains represented by the Sicilian/Calabrian sectors of the collisional/subduction belt and the foreland basement domain. In the south, the Pelagian block and Ionian Sea are separated by a Mesozoic lithospheric boundary. The NNW–SSE-striking Hyblean–Maltese Escarpment fault system (HMEFS; e.g., Doglioni et al. 2001) represent the continental and oceanic foreland of the underthrusting Nubian plate (Roure et al. 1990; Ben-Avraham and Grasso 1991). The orogenic zone includes the Sicilian–Maghrebian Chain and the Calabro-Peloritan Arc; with the former a S–SE verging thrust belt considered to be the eastern prolongation of the North African Chain, and the latter extending from southern Calabria to the north-eastern part of Sicily (overthrusting the Maghrebian domain) and hosting the innermost geological units formed from the deformation of the European margin domains (e.g., Bouillin et al. 1986). The current tectonic setting of the Calabro-Peloritan Arc, which connects the NW–SE-trending southern Apennines with the WSW-striking Maghrebian thrust zones, has been closely related to the opening of the Tyrrhenian Sea, associated rollback of the Ionian slab, and to ESE-ward drift of the Calabro-Peloritan massif (Malinverno and Ryan 1986; Billi et al. 2011).

Evidence for Ionian slab rollback has been found in tomographic images (e.g., Chiarabba et al. 2008; Neri et al. 2009; Scarfì et al. 2016) and in the occurrence of intermediate and deep earthquakes (~50–500 km) that point to a NW dipping and sinking volume beneath the Calabrian Arc and southern Tyrrhenian Sea. This structure can be considered as the residual of an older slab extending >1000 km from northern Italy to the Maghrebides (e.g., Spakman and Wortel 2004).

Recent studies (e.g., Gutscher et al. 2016; Polonia et al. 2016; Scarfì et al. 2016) have shown that the NW–SE-trending lithospheric-scale tear zone through the Aeolian Islands, north-eastern Sicily, and the Ionian Sea can be explained, not only by the subducted slab, but also by regional transtensional fault systems caused by the structural complexity of the crustal boundaries. These structures may have a primary role in driving the geodynamics of the Central Mediterranean and likely influence the evolution and activity of Mt. Etna.

### Mt. Etna Volcano

Mt. Etna, one of the largest volcanoes in Mediterranean region, is located just north of the Hyblean plateau margin (Fig. 1b). The volcano, which has a peculiar structural position and geochemical features, is located on an accretionary prism close to the lithospheric discontinuity of the Ionian subducting plate (e.g., Doglioni et al. 2001). Erupted products are characterized by intraplate affinity with the southern Apennine. Activity at Mt. Etna began at ~500 ka, and after an initial phase characterized by tholeiitic products, has produced slightly Na-alkaline volcanic products with a prevalence of hawaiitic lava emissions. Successive episodes built different volcanic edifices, which have shifted westwards, with present-day activity focused on Mongibello. Late Pleistocene and Holocene Mongibello activity (<30 ka) has been characterized by recurrent, significant explosive activity (paroxysmal eruptions) that formed currently recognizable calderas (e.g., Ellittico caldera at ~15 ka and Piano caldera in 122 B.C.). In recent centuries, volcanic activity has mainly been characterized by lava effusions, lasting from hours to several months or years. Explosive activity has remained mild, ranging from semi-persistent at summit vents to sporadic at lateral vents.

The volcano structure is surrounded by three main regional structures: (1) the Hyblean Foreland to the south, which belongs to the African plate (Lentini et al. 2006); (2) the Apennine–Maghrebian chain to the north and west; and (3) the extensional Ionian basin to the east, which originated during the middle–late Mesozoic (Catalano et al. 2001).

An extensional regime characterizes the eastern part of the volcano, while the western part is dominated by the regional ~N–S compressive regime associated with the Eurasia-Africa plate collision (Monaco et al. 2005).

A number of alternative settings have been proposed to account for the development of the volcanic edifice, including a hot spot origin (Tanguy et al. 1996), asymmetric rifting (Continisio et al. 1997), dislocation between the ‘Malta-Sicilian block’ and the Ionian Basin (Gillot et al. 1994), and the intersection tectonic features (Di Geronimo et al. 1978; Lanzafame and Bousquet 1997; McGuire et al. 1997). Recently, Schellart (2010) suggested that volcanic activity at Mt. Etna may be related to decompression melting of upper mantle material flowing around the southern Ionian slab edge to accommodate east-directed rollback of the Ionian slab that resumed in the late Miocene.

### Aeolian Islands

The Aeolian Arc is a volcanic structure, ~200 km in length, located on the internal margin of the Calabrian–Peloritan forearc region and affected by Late Quaternary extensional tectonics and uplift. The arc consists of seven subaerial volcanic islands (Alicudi, Filicudi, Salina, Lipari, Vulcano, Panarea, and Stromboli), emplaced on continental crust with a thickness of 15–20 km. The volcanic activity is dated between 1.3 Ma and the present. Volcanic products are mainly characterized by calc-alkaline, high-K calc-alkaline, shoshonitic, and alkaline potassic associations.

The origin of this back-arc archipelago relates to Ionian lithosphere subduction beneath the Calabrian Arc (e.g., Barberi et al. 1974; Mantovani et al. 1996) and a postsubduction extensional deformation (Beccaluva et al. 1985; Westaway 1993; Ventura et al. 1999; De Astis et al. 2003).

Active volcanism is concentrated on the north-eastern islands (e.g., Stromboli). In addition, the islands can be categorized into three sectors distinguished by regional stress fields: (1) the Western sector (Alicudi and Filicudi islands), dominated by a WNW–ESE-striking fault system (Sisifo–Alicudi System); (2) the Central sector, containing NNW–SSE-striking faults (Tindari–Letojanni fault system) that affect overlying volcanoes, which align along a NNW–SSE-striking structural depression (e.g., Barberi et al. 1994, Cultrera et al. 2017); and (3) the eastern sector (Panarea and Stromboli islands), characterized by prevailing NNE–SSW to NE–SW-striking fault systems.

## Experiment, Data, and Algorithms

### Previous Seismic Experiments

Since the early 1970s, the crustal structure of Sicily and its surrounding areas have been the focus of several seismic experiments. These studies can be divided into three phases according to their period and results. During the first phase (1968–1986), eight crustal refraction/wide angle R/WAR profiles were performed on-land, but were mainly characterized by poor acquisition geometry and low-quality data. The second phase (1991–1995) coincided with the CROP Sea projects I–II and with the CROP LAND SEA Group, who performed several offshore-onshore structural profiles. The third phase (1993 to the present) has included deep seismic surveys that have mainly focused on offshore eastern Sicily. However, in general, the eastern Sicily region has been less studied and the available data are mainly limited to old DSS profiles that provided no new information on the structure of the crust or on the transition zone between the Peloritanian block and the Apennine–Magrebide chain.

The first deep seismic experiment at Mt. Etna was performed in 1977 (Colombi et al. 1979). Using data obtained during this experiment, Sharp et al. (1980) modelled the structure of Mt. Etna and suggested that a 15–25-km-depth low-velocity triaxial ellipsoid body extending beneath the entire volcanic area represents a potential magma chamber. Since the 1990s, seismological studies have progressively improved our knowledge of the structure beneath Mt. Etna; in particular, through tomographic inversions of P- and S-waves, both in velocity and attenuation (e.g., Hirn et al. 1991, 1997; Cardaci et al. 1993; De Luca et al. 1997; Laigle et al. 2000; Chiarabba et al. 2000, 2004; Patane et al. 2002, 2003, 2006; Martínez-Arevalo et al. 2005). A detailed review of these studies is presented in Ibáñez et al. (2016a, b); however, it is noteworthy that none revealed the presence of the above-mentioned deep low-velocity region beneath the volcano.

The Aeolian volcanic environment has also been studied through high-resolution passive seismic tomography in using velocity and attenuation (e.g., Chiarabba et al. 2008; Di Stefano et al. 2009), and the results of this have shown direct relationships between volcanic structures and low-velocity and high-attenuation bodies.

### TOMO-ETNA: A Joint Passive and Active Seismic Experiment

**Fig. 2:**
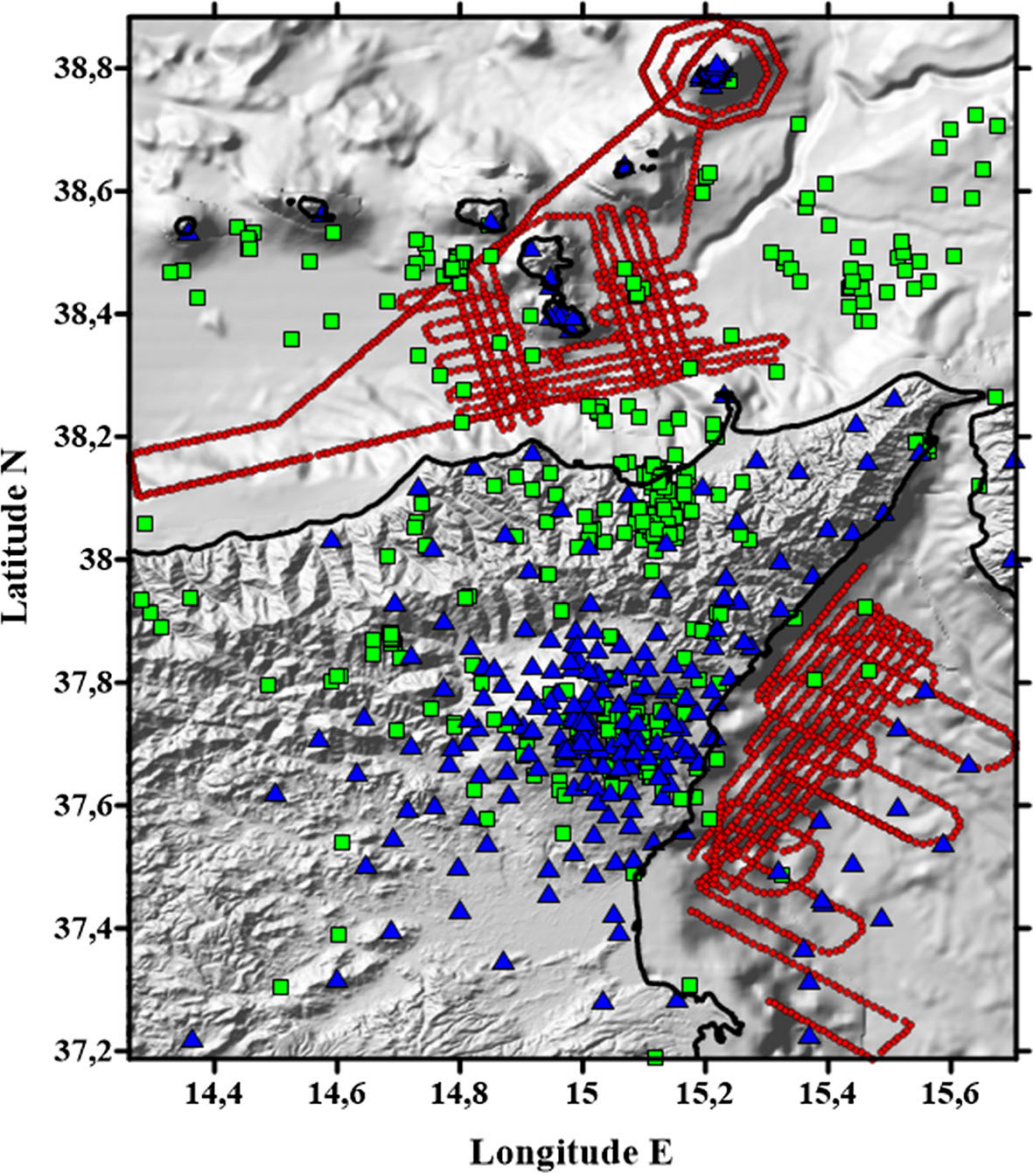
Map of the study region showing seismic station distribution (blue triangles), the epicentral positions of passive seismic sources (green squares), and the locations of active seismic sources (red dots)

The TOMO-ETNA experiment was performed in 2014 under the frameworks of the EC-FP7 MED-SUV and EUROFLEET2 MED-SUV.ISES projects (Coltelli et al. 2016; Ibáñez et al. 2016a, b). The experiment aimed to investigate the inner structure of Mt. Etna and the surrounding areas, including the Maghrebian Chain, the HMEFS, the southern Calabria zone, and the Aeolian Islands archipelago, by using multidisciplinary approaches. One of the most relevant and innovative aspects of this project was a joint inversion of active and passive seismic data aimed at relating volcanic processes to shallow and deep structures. For this purpose, data were collected over a large area (300 × 300 km), including both terrestrial and marine territories (Fig. 2). The dataset is also adequate for performing 2-D and 3-D attenuation tomography (in preparation), while ~1410 km of marine seismic reflection profiles were acquired to image in detail the seismic-stratigraphic and structural setting of the crust down to/approaching the limit of the Moho discontinuity beneath the Ionian and Tyrrhenian seas (Coltelli et al. 2016; Firetto-Carlino et al. 2016). Finally, high-resolution bathymetry, magnetic surveys, and remotely operated vehicle (ROV) imaging were performed (e.g., Cavallaro et al. 2016).

In this study, we primarily used data from the active seismic experiment performed using energy sources at sea. These signals were recorded by portable seismic stations deployed on land and on the seafloor (Fig. 2) together with stations of a permanent seismic network belonging to the Italian Institute of Geophysics and Volcanology-INGV (Barberi et al. 2016; Ibáñez et al. 2016b). Seismic signals were generated using air guns on two oceanographic research vessels, the Spanish vessel ‘Sarmiento de Gamboa’ and the Greek vessel ‘Aegaeo’ (Coltelli et al. 2016). This experiment represents the most complex performed in the region and probably one of the most complete and complex active seismic experiments ever performed in a region combining volcanic and tectonic environments (Ibáñez et al. 2016a, b).

The success of this experiment is evidenced by the large number of additional scientific works that have utilized the data produced, including scattering analysis (Zieger et al. 2016), 3-D seismic array analysis (Zuccarello et al. 2016), and the study of how geophysical marine campaigns impact on aquatic mammals (Monaco et al. 2016). Furthermore, the establishment of long-term research programmes based on the results is expected, including 2-D and 3-D seismic attenuation tomography with separation of scattering and intrinsic effects (e.g., Prudencio et al. 2013a, b, 2015a, b, c, 2017; Del Pezzo et al. 2016); identification of seismo-volcanic signals (e.g., Benítez et al. 2007; Ibáñez et al. 2009; Cortés et al. 2014; 2016); nonlinear relocation of seismicity (e.g., Díaz-Moreno et al. 2015); study of tremor, long period (LP) events, and explosion quake source inversion (e.g., La Rocca et al. 2000, 2004; Saccorotti et al. 2004; Petrosino et al. 2011); analysis of explosion wave-field properties (e.g., Palo et al. 2009; De Lauro et al. 2012); shear waves splitting (e.g., Martínez-Arévalo et al. 2003; Bianco and Zaccarelli 2009); analysis of scattered seismic wave fields (e.g., Del Pezzo et al. 1997; De Barros et al. 2012); and analysis of receiver functions (e.g., Lodge et al. 2012).

#### Seismic Instruments

In total, 267 seismic stations were used for this experiment (Fig. 2). On-land portable seismic stations were provided by the Geophysical Instrument Pool Potsdam (GIPP), Germany (Ibáñez et al. 2016a, b). In total, 90 DATA-CUBE3 recorders equipped with triaxial short-period seismometers PE-6/B 4.5 Hz or Mark L-4C-3D (with a 4.5–150 Hz flat response range), and 17 broadband (BB) portable seismic stations with Earth-Data PR6-24 recorders and three-component Nanometrics Trillium Compact seismometers (120 s to 100 Hz response range) were employed. For each, the sampling rate was set at 100 Hz.

The temporal network took advantage of several OBS stations, including 15 provided by the Unidad Tecnológica Marina (CSIC-UTM), Barcelona, Spain. These LC SP 4 × 4 IGPP-SIO-UCSD instruments include a HighTech HTI-90-U hydrophone. The geophone is a short-period Sercel L28 model, with a natural frequency of 4.5 Hz and a sample rate of 200 sps. The data logger and electronics are BART ORE Offshore software sampling at 200 sps. In addition, the INGV ‘Osservatorio di Gibilmanna’ in Palermo (Italy) provided two types of OBS: i) ten short-period SM/6 Geophones with a natural frequency range of 4.5–140 Hz and with a 250-sps sampling rate; and ii) two Broadband Güralp CMG40T-OBS sensors with a natural frequency ranging between 60 s and 100 Hz and with a sample rate of 100 sps.

Additionally, we used the data recorded by 133 seismic stations of the INGV permanent seismic network operated by the ‘Osservatorio Etneo’ at Catania (Italy). Seismic stations in this network comprise broadband three-component Nanometrics Trillium seismometers.

#### Seismic Sources

Active seismic sources were provided by the Spanish oceanographic vessel ‘Sarmiento de Gamboa’ and consisted of 16 air guns deployed along a two-line array, 10 m below sea level (b.s.l.). The volumes of the different guns were 520 × 4, 380 × 4, 250 × 4, and 150 × 4 cubic inches (cu.in), producing a total volume of 5200 cu.in. They were spaced at 1-m intervals along the line, with a 9-m interval between the lines. In total, more than 16,500 shots were fired (Fig. 2).

Besides the active seismic sources, a total of 452 earthquakes (Fig. 2) were selected from the INGV database for the period 1 June to 30 November 2014 (Barberi et al. 2016). All of these seismic events were recorded on at least three seismic stations of the permanent INGV network, and inside the region selected for this study. The magnitude (Ml) of these events ranged from 0.3 to 3.8, and the focal depths were from 1.5 km above sea level (Mt. Etna edifice) to 215 km b.s.l. (subduction structure below the Aeolian Islands). The selected period was chosen in order to produce a tomographic snapshot of the regional structure.

### P-Wave First Arrival Picks

In this study, we applied automatic procedures for P-phase determination based on the Adaptive Multiband Picking Algorithm (AMPA) developed by Alvarez et al. (2013). This software is characterized by its adaptability. It was originally developed for passive-source P-first onset determination; however, for the TOMO-MT. ETNA dataset, we re-configured the algorithm for both passive and active seismic-source picking. The AMPA strategy determines P-wave onset times for signals strongly affected by non-stationary noise and/or with low signal-to-noise ratio (SNR). This procedure is performed in two main steps: (1) multiband envelope detection and noise reduction, eliminating contributions below band-dependent envelope thresholds; and (2) enhancement of signal envelopes and durations corresponding to P-phase arrivals.

Calibration of AMPA parameters depends on the signals to be picked and usually starts by defining the bands of analysis (lower and upper frequencies), including the number of sub-bands (hereafter referred as ‘*k*’). For the TOMO-MT. ETNA data, a 4–12 Hz band was selected, even during a pre-filtering stage, for both active and passive data. This frequency band was selected according to the characteristics of the active seismic signals (Ibáñez et al. 2016b) and to avoid any contamination from background volcanic tremor and noise (e.g., Saccorotti et al. 2004; Ibáñez et al. 2009). Moreover, considering the length of the expected events (active and passive sources), we set the number and length of the optimum filters. For this study and after several empirical calibrations, we defined three filters (200, 100, and 50 samples) for sampling frequencies of 100 Hz.

Quality parameters and threshold can also be defined during the AMPA procedure. In this study, we implemented three parameters: (1) *Z*_max_ represents the similarity of observed and theoretical waveforms. A minimum *Z*_max_ value of 6 (the higher the value the better); (2) SNR of 2; and (3) Δ_time_ of <0.5 s between successive events (García et al. 2016). These threshold values ensured high-quality data. The suitability and success of this automated procedure is illustrated in “Appendix 1”.

The final P-wave first arrival database comprises 184,797 active P-phase travel times and 11,802 passive P-phase travel times.

### Tomographic Inversion: PARTOS Software

A tomographic algorithm for joint inversion of active and passive seismic data has recently been implemented in new software called PARTOS (Díaz-Moreno et al. 2016). The code permits inverting P- and S-wave velocities and *V*_p_/*V*_s_ ratios for both active and passive data leading to a joint 3-D velocity model. PARTOS was developed alongside the TOMO-MT. ETNA experiment and has been intensely tested using several synthetic tests (see “Appendix 3”).

In PARTOS, passive data source locations are conducted in three steps: (1) absolute preliminary location by applying the Nolet (1981) analytical formulae for ray tracing in a 1-D model; (2) 3-D location in the P and S velocity models using a ray-bending method based on the Um and Thurber (1987) time minimization principle; and (3) source parameters, station corrections, and P and S anomaly calculations. Unreliable locations are then discarded by identifying outlier time residuals. The localization step is carried out iteratively for a pre-established number of iterations. At each iteration, an updated 3-D velocity model is used for the localization.

Active data do not require relocation; therefore, the initial step becomes the ray tracing, which was carried out using the same ray path method algorithms. Calculations are done using the regular 3-D ray-bending tracer between the source and the station. For TOMO-MT. ETNA, offshore shooting was performed on the sea surface; therefore, the ray was computed for the entire path taking into account the water layer with a constant *V*_p_ of 1.44 km/s. Once ray tracing is performed, active and passive data are merged into a single joint dataset that is used for further calculations.

Grid construction is carried out by constructing a mesh of nodes according to the ray density. The distribution of nodes along the studied volume depends on the density of both active and passive rays. Velocity anomalies are linearly interpolated between nodes. Usually, the grid spacing is set smaller than the expected resolution so that every restored anomaly will be based on several nodes. This helps to avoid the dependency of results on grid configuration. To further reduce this effect, we performed the inversion for several grids with different orientations (four grids with orientations of 0°, 22°, 45°, and 67°) and then averaged them in one 3-D model using a regular mesh. The grid is constructed only once during the first iteration. In the following iterations, velocity anomalies are updated in the same nodes.

To perform tomographic inversion, we computed one matrix that includes pairs of active and passive data. The matrix elements responsible for the velocity distributions are the first derivatives *A*_*ij*_ representing time deviation along the *j*th ray due to velocity variations in the *i*th node (Eq. 1):

1 $$A_{ij} = \mathop \int \limits_{\gamma }^{ }\Delta g_{j} \left( \gamma \right){\text{d}}S/\Delta \sigma_{j}$$

where $\Delta g_{j}$ represents the slowness perturbation at the point of the ray caused by slowness anomaly $\Delta \sigma_{j}$ at the *j*th node (Koulakov et al. 2006). Values are computed by numerical integration along the ray paths derived after the location steps (for passive data) or after the ray-tracing step (for active data). The matrix inversion is performed using the LSQR algorithm (Paige and Saunders 1982).

#### Inversion Parameter Selection

Damping and smoothing parameters, together with the grid dimensions and initial 1-D velocity models, were varied to check the impact on the results. Damping parameters, although controlling the extent of the anomalies, did not dramatically impact on the results, with values of 0.8–1.2 leading to a final 1.0 damping value. In contrast, smoothing values significantly affected the quality of the resulting images. The initial 1-D velocity model, which controls the depth of the earthquakes, was found to be crucial for achieving realistic results. Taking into account the full results of these tests (Appendix 1), including all of the tested parameters and both the lowest residuals and quality of the images, an optimal configuration was selected (Table 1).

**Table 1:** Parameter settings tested for Region 1

Dataset	1-D vel. model	Smoothing	Weights	Test	Residuals (ACT/PAS)	Number of Rays (ACT)	Number of rays (PASS)	Name
Active	1	1	1	1	0.167	153,750	0	Model_01
Passive	1	1	1	1	0.243	0	11,802	Model_02
Joint	1	1	1	1	0.180/0.246	7543	11,802	Model_03
Joint	1	1	1	2	0.166/0.296	153,318	10,227	Model_04
Joint	1	1	1	3	0.158/0.304	158,174	5366	Model_05
Joint	2	1	1	2	0.166/0.291	153,653	10,249	Model_06
Joint	3	1	1	2	0.173/0.289	151,808	10,224	Model_07
Joint	2	1	1	2	0.166/0.289	153,752	10,249	Model_08
Joint	2	1	2	2	0.168/0.279	154,338	10,249	Model_09
Joint	2	1	3	2	0.168/0.279	154,338	10,249	Model_10
Joint	2	1	4	2	0.177/0.279	154,338	10,249	Model_11
Joint	2	2	4	2	0.170/0.279	154,338	10,249	Model_12
Joint	2	1	5	2	0.177/0.251	153,706	10,249	Model_13
Joint	4	1	5	2	0.171/0.252	155,326	10,241	Model_14
Joint	4	2	5	2	0.167/0.246	155,614	10,241	Model_15
Joint	4	3	5	2	0.175/0.256	154,793	10,241	Model_16
Joint	5	1	5	2	0.178/0.289	151,613	10,183	Model_17
Joint	5	2	5	2	0.176/0.280	152,017	10,183	Model_18
Joint	5	3	5	2	0.181/0.290	151,313	10,183	Model_19
Joint	6	1	5	2	0.179/0.265	152,319	10,152	Model_20
Joint	6	2	5	2	0.177/0.271	152,656	10,152	Model_21
Joint	6	3	5	2	0.182/0.267	151,928	10,152	Model_22
Joint	7	1	5	2	0.178/0.265	152,158	10,172	Model_23
Joint	7	2	5	2	0.176/0.262	152,452	10,172	Model_24
Joint	7	3	5	2	0.181/0.268	151,737	10,172	Model_25
**Joint**	**7**	**4**	**5**	**2**	**0.177/0.264**	**152,319**	**10,172**	**Model_26**
Joint	7	4	5	2	0.178/0.266	151,304	10,172	Model_28

Bold text denotes the selected configuration

## Results: Seismic Tomographic Images

Based on our data quality tests (Appendix 1), we divided our study region into three complementary areas and estimated optimum grid sizes for each. Region 1 (the first inversion) covered the whole study area (300 × 300 km), including NE Sicily and both volcanic areas. Region 1 was inverted using a grid spacing of 6 km in the horizontal and 2 km in the vertical. Region 2 covered the Aeolian Islands archipelago (126 × 126 km) and was inverted with 4-km horizontal and 2-km vertical grid spacings. Region 3 covered the Mt. Etna volcanic system (60 × 60 km) and was inverted using grid spacings of 2 km in both the horizontal and vertical.

**Fig. 3:**
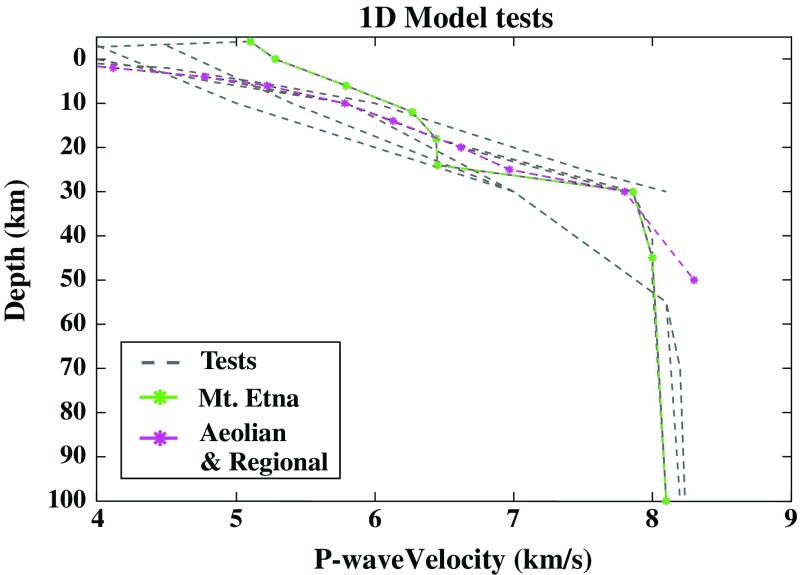
Starting velocity models (1-D). Grey dashed lines represent different tests; pink line marks the starting 1-D velocity model for both Region 1 (whole study area) and Region 2 (Aeolian Islands archipelago); the green line marks the starting 1-D velocity model for Region 3 (Mt. Etna)

Results of the tomographic inversions are 3-D distributions of absolute P-wave velocity starting from a preferred one-dimensional (1-D) velocity model (Fig. 3). Initial model selection is critical to obtaining reliable final results. We tested a range of initial 1-D models that were consistent with a priori knowledge of regional tectonics, geology, and the results of previous seismic tomography investigations (e.g., Alparone et al. 2012; Neri et al. 2009). From this starting model, it was possible to derive 3-D distributions of anomalies in P-wave velocities. These anomalies were obtained as the difference of the final model and the mean expected velocities of each layer.

In our analyses and discussion of results, we focused our attention on the anomaly distribution, rather than on absolute velocities, on the basis that: (a) 3-D anomaly distributions are mostly quasi-independent of the starting 1-D model; (b) many tectonic and volcanic structures are better illuminated using these distributions; and (c) since each analysed region was inverted with its own 1-D velocity model, joint discussion was more appropriate.

Different velocity model variations were tested in order to find the best compromise between a priori information and simplicity, thus ensuring the optimal starting set up (Fig. 3). For regions 1 and 2, we selected a 1-D model derived from that proposed by Neri et al. (2009) for the whole of Sicily, as it best averaged the different major geological structures and rock compositions (Fig. 3). For Region 3, a more specific velocity model was required to account for the specifics of the Mt. Etna volcanic system.

### Joint Tomography of Region 1 (Whole Region)

**Fig. 4:**
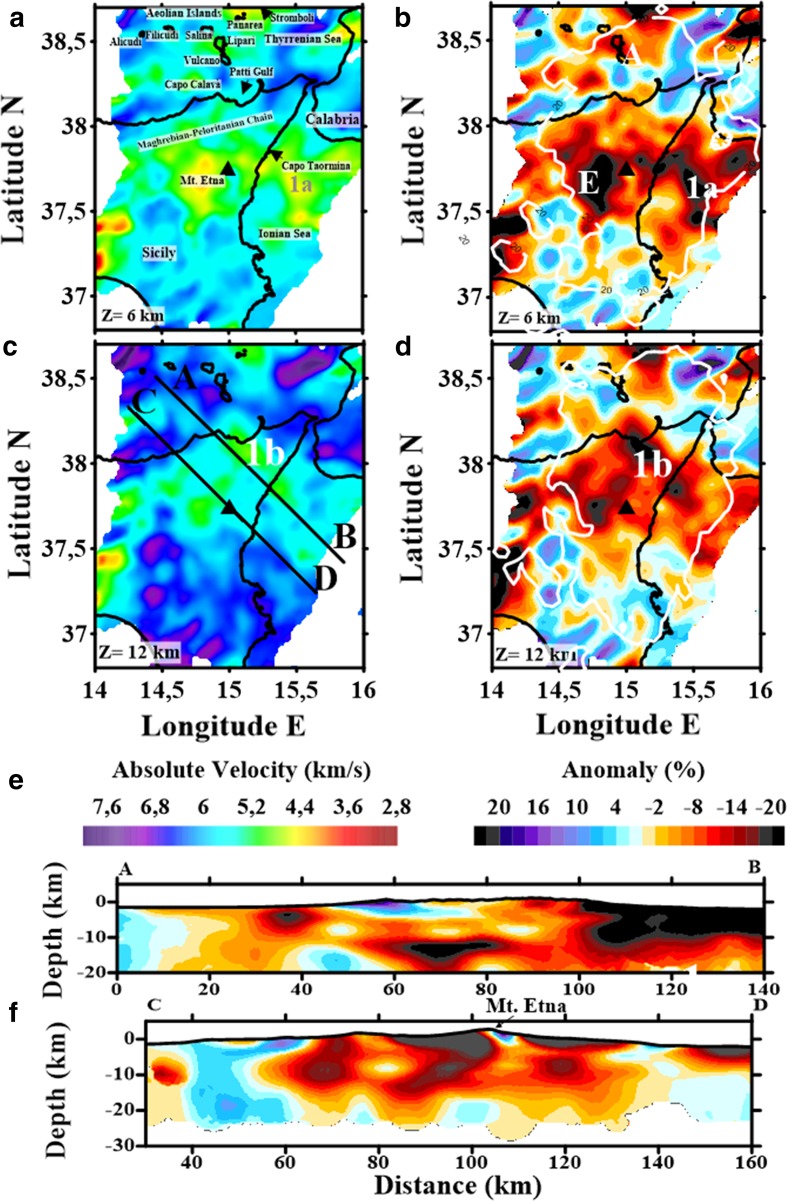
**a** P-wave absolute velocity; and **b** P-wave velocity anomaly maps for Region 1 at 6 km depth; **c** P-wave absolute velocity map for Region 1 at 12 km depth; **d** P-wave velocity anomaly map for Region 1 at 12 km depth. Black triangle represents Mt. Etna; **e** P-wave velocity anomaly vertical section A–B; **f** P-wave velocity anomaly vertical section C–D. Labels are as follows: (1a) NW–SE low-velocity anomaly (up to 8–10 km depth); (1b) NW–SE low-velocity anomaly (10–14 km depth); (A) Aeolian Island archipelago (Region 2); and (E) Mt. Etna Volcanic system (Region 3)

Figure 4 shows the final tomographic images for Region 1 (i.e. the whole study region). The obtained models include several shallow high- and low-velocity anomalies with amplitudes of up to ±24%. These models have a maximum resolved depth of 15 km b.s.l., according to our tests (Appendix 1). To help with the interpretation of images and more easily link them with tectonic or volcanic features, we selected two layers (6 and 12 km depth; Fig. 4a–d) and one vertical section (Fig. 4e).

Anomaly E (the volcanic edifice/system of Mt. Etna) was easily identified (Fig. 4b). This region shows a lower mean velocity anomaly than the methamorhic Maghrebian–Peloritanian chain of northern Sicily, with the difference reaching up to −24%, producing a strong contrast with surrounding areas. A similar low-velocity pattern was observed for the volcanic arc of the Aeolian Islands; however, an additional low-velocity anomaly on the south-western margin of the study area (Fig. 4b) was beyond our resolution, which prevented us for making interpretation about its nature.

Two other significant anomalies were observed for Region. Anomaly 1a is a NW–SE velocity contrast that extends ~60 km from Capo Taormina towards the Ionian Sea (Fig. 4a, b). This low-velocity anomaly is clearly visible from 4 km b.s.l. up to 8–10 km b.s.l. It presents absolute velocity values of 4.4 km/s, which is in contrast to surrounding values (~5.2 km/s). Furthermore, this represents a −20% anomaly from the starting velocity model. Anomaly 1b is a deepening extension of anomaly 1a, extending NW to a depth of ~14 km b.s.l., reaching the coast of Vulcano Island. In this case, the absolute velocity is 5.5 km/s for the anomaly and >6 km/s for the surrounding area (Fig. 4c), creating a difference of −20% (Fig. 4d). Figure 4e shows a vertical section that images the geometry of this at depth.

### Joint Tomography of Region 2 (Aeolian Islands)

**Fig. 5:**
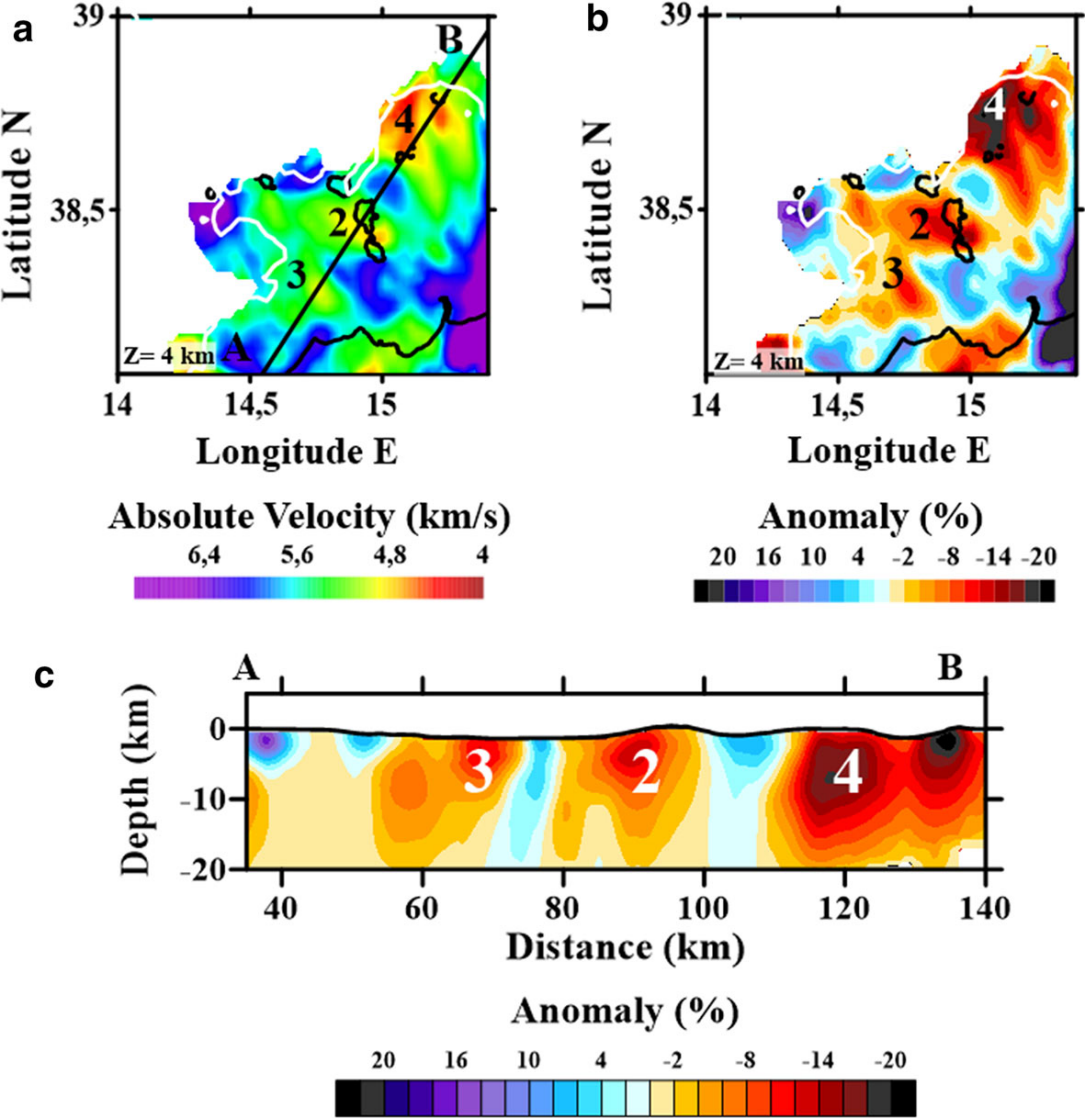
**a** P-wave absolute velocity; **b** P-wave velocity anomaly maps for Region 2 at 4 km depth; **c** P-wave absolute velocity and velocity anomaly vertical section A–B. Labels are as follows: (2) WNW–ESE low-velocity anomaly (2–14 km depth); (3) NW–SE low-velocity anomaly (2–8 km depth); (4) circular low-velocity anomaly below Stromboli-Panarea region (2–14 km depth)

The tomographic inversion of the Aeolian Islands archipelago (Region 2; Fig. 5), which covered an area of 126 × 126 km with a grid spacing of 4 km (horizontal) by 2 km (vertical), extended to a depth of 24 km b.s.l. (Appendix 1). Although the resolution of this area is not as good as that for the other regions, the images obtained still allow us to better define some of the major features highlighted in the Region 1 inversion.

In addition to the previously identified anomalies (1a and 1b), we observed Anomaly 2, which is characterized by absolute velocities of 4.5 km/s at 4 km b.s.l., significantly lower than mean Thyrenian Sea velocities at the same depth (Fig. 5a) and reaching −15% of the starting velocity model (Fig. 5b). This structure, striking approximately WNW–ESE and extending between Filicudi and the Vulcano–Lipari complex, has previously been identified as the Sisifo-Alicudi Fault System (SAFS). SAFS is a dextral strike-slip motion fault system that accommodates the compression of the westernmost part of the Aeolian Islands archipelago (Barreca et al. 2014). The eastern limit of this belt juxtaposes the Aeolian–Tindari–Letojanni fault system (ATLFS) near Vulcano Island, while the westernmost limit coincides with the compressional W–E belt.

Anomaly 3 occurs at 4–8 km depth and represents another velocity discontinuity (low/high anomaly contrast) extending NW–SE offshore of Capo Calavà (Fig. 5). Finally, Anomaly 4 is characterized by a large volume low *V*_p_ anomaly (−20%) extending between Stromboli and Panarea (Fig. 5). Its location on the edge of the well-resolved area makes confident interpretation challenging; however, there is a broad agreement with the results of other studies (Piromallo and Morelli 2003; Montuori et al. 2007; Chiarabba et al. 2008; Caló et al. 2009; Scarfì et al. 2016), who argued for the accumulation of a significant volume of partial mantle melt the feeding present-day activity of Tyrrhenian volcanoes.

### Joint Tomography of Region 3 (Mt. Etna)

**Fig. 6:**
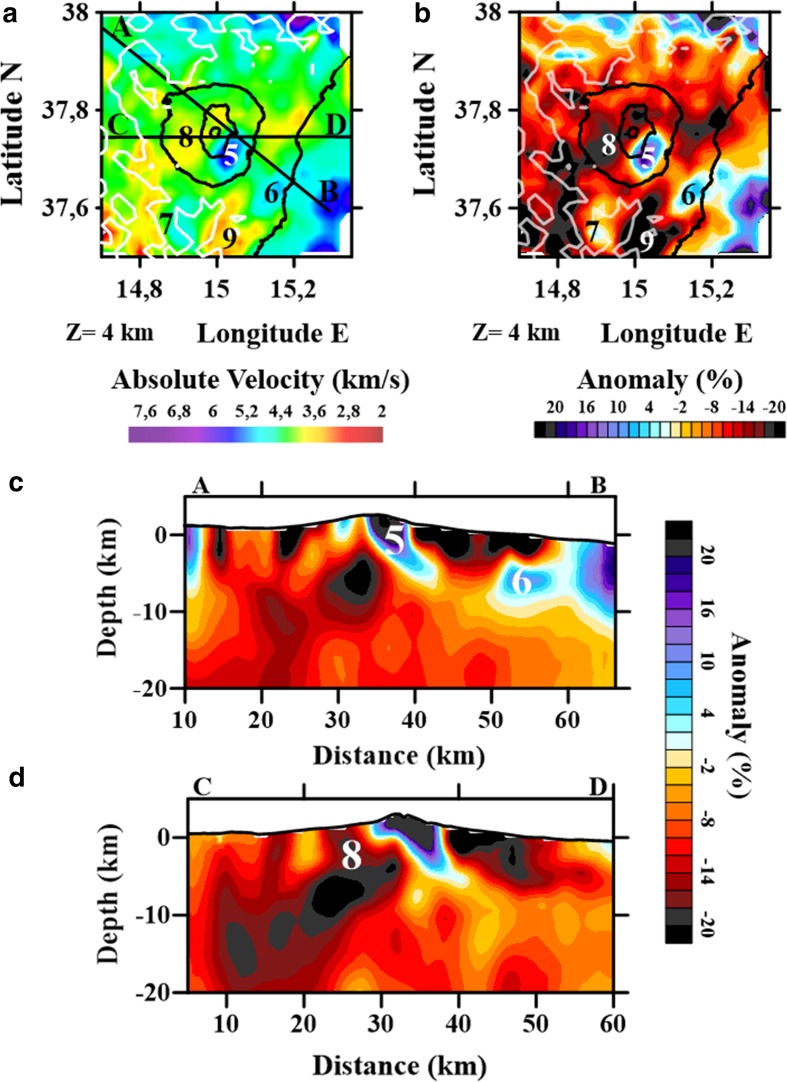
**a** P-wave absolute velocity; **b** P-wave velocity anomaly maps for Region 3 at 4 km depth; **c** P-wave velocity anomaly vertical section A–B; **d** P-wave velocity anomaly vertical section C–D. Labels are as follows: (5) rounded high-velocity anomaly slightly SE of the central craters; (6) rounded high-velocity anomaly located on the coast; (7) rounded high-velocity anomaly located SW of the central craters; (8) rounded low-velocity anomaly located W of the central craters; (9) rounded low-velocity anomaly located S from central

The tomographic inversion of the Mt. Etna region, which had a grid size of 2 × 2 km, extended to a depth of 14 km b.s.l. (Appendix 1). The results showed the presence of several high- and low-velocity anomalies (Fig. 6); however, in our analyses we focused on those anomalies considered robust according to our data quality tests (Appendix 1), including a high density of seismic rays used to invert the travel time misfit.

Of the high-velocity anomalies, Anomaly 5 is located towards the south-east, underneath the central craters and is a high-velocity anomaly widely known as the high-velocity body (HVB; e.g., Patanè et al. 2006; Alparone et al. 2012). Anomaly 6 is located in the south-eastern sector of Mt. Etna area, close to the coast. This volume was clearly resolved between 4 and 14 km depth, where it disappears or collides with a larger high-velocity anomaly offshore and to the east (Fig. 6a–d). Finally, Anomaly 7 located south-west of the volcanic edifice. This high-velocity structure, which has not previously been observed, may be correlated with a high-density anomaly up to 6 km b.s.l., as indicated by preliminary gravimetric analyses (J. Fernández, personal communication).

Region 3 contained two low-velocity anomalies. Anomaly 8 is located to the west of the central craters, in the same sector where Aloisi et al. (2002) also highlighted a low-velocity volume, which has been associated with the presence of a magmatic reservoir and correlated with anomalous b values revealed by Murru et al. (1999). Anomaly 9, which has not previously been observed, is located to the south of Mt. Etna and contains absolute velocity values of around 3.6 km/s at 4 km b.s.l., representing an anomaly of up to −20%.

## Discussion

Investigating the 3-D crustal velocity structure of north-eastern Sicily, including Mt. Etna and the Aeolian Islands archipelago, has allowed us to develop a detailed and complete regional seismotectonic framework that highlights a spatially heterogeneous tectonic and volcanic regime. This is the first time that active and passive seismic sources have been used together to obtain a 3-D image of regional velocity, and it is also the first time that data from a dense seismic network deployed on both land and the sea bottom have been used.

In the present work, images obtained from inversion of the whole region were complementary to those of the smaller sub-region, despite lower resolution, and actually provided information to greater depths. For example, the inversion of the Mt. Etna region (i.e. that with the highest resolution) provided to around 10 km b.s.l., while the inversion of the whole region extended to 15 km below the volcanic structure. By combining both images, we were able to better constrain the regional crustal structure.

### North-Eastern Sicily–Peloritan Region–Ionian Sea

Figure 4 shows a major discontinuity striking roughly NW–SE, extending from the Gulf of Patti to the Ionian Sea. Although resolution is limited outside of this area, the discontinuity may correspond to the ATLFS, a regional deformation belt. Rosenbaum et al. (2008) suggested the presence of the ATLFS by interpreting a gap within the subducting lithosphere at 100–150 km deep along the Salina, Lipari, and Vulcano alignment (i.e. along the Tindari–Letojanni fault system). This represents the western boundary of the Ionian slab below the Calabrian Arc, whose extension in the western zone of the Ionian Sea has shown in recent analyses of new high-resolution seismic profiles and detailed morpho-bathymetry of the seafloor (e.g., Gutscher et al. 2016; Polonia et al. 2016).

Our results indicate that the ATLFS could be associated with a NW–SE sharp velocity contrast (Fig. 4e; A–B cross section). This velocity contrast is well evidenced in the Ionian area oceanic crust from shallower layers (4 km) up to the resolution limit (14 km) and in north-eastern Sicily, where it is better detected in the intermediate crust (>10 km b.s.l.). This could be explained using a more complex structural framework that characterizes the shallow continental crust, as suggested by Barreca et al. (2014) and Cultrera et al. (2017). These findings are in agreement with the features shown by Scarfì et al. (2016), who correlated tomographic and seismic patterns with the existence of a sub-crustal structure.

We were unable to identify confirm a low-velocity region (i.e. potential magma chamber) at great depth below Mt. Etna (Fig. 4f; C–D cross section). Furthermore, the potential feeding channel for providing molten material to the volcano is not clearly marked in our results. Using the HVB as a reference, we observed some possible low-velocity anomalies from surface to depth to both sides of the structure, which could be interpreted as magma ascent channels.

### Aeolian Islands Archipelago

Around the Aeolian Islands were observed a number of significant tomographic features. A velocity contrast striking WNW–ESE and extending between Filicudi and the Vulcano–Lipari complex (number 2 in Fig. 5) could be connected to the SAFS (e.g., Barreca et al. 2014). However, this low-velocity area could also reflect the volcanic nature of the overlying islands (Vulcan and Lipari), as evidenced by its similarity to another low-velocity anomaly extending beneath Panarea and Stromboli (number 4 in Fig. 5), which has been interpreted as the probable location of magma accumulation beneath the Tyrrhenian volcanoes (Piromallo and Morelli 2003; Montuori et al. 2007; Chiarabba et al. 2008; Caló et al. 2009; Scarfì et al. 2016).

Finally, another low-velocity anomaly (number 3 in Fig. 5) is associated with the Patti Gulf-Capo Taormina-Ionian Sea alignment along a NW–SE to NNW–SSE direction. This anomaly is considered as a discontinuity that marks the current southern termination of the Ionian subducting lithosphere. Its activity in north-eastern Sicily and the Ionian Sea has been recognized at sub-crustal depths by tomographic images, seismic event distribution, and focal mechanism kinematics (e.g., Scarfì et al. 2016; Polonia et al. 2016). At shallower depths, the possible lack of a NW–SE velocity discontinuity has been interpreted as a lithospheric-scale tear fault, characterized by a transtensional complex with NW–SE and NE–SW fault systems and topographic uplift (Faccenna et al. 2011; Scarfì et al. 2016; and references therein).

### Mt. Etna Volcano

Mt. Etna presents the highest number of velocity contrasts, which is consistent with the complex nature volcanic environments. From the structures identified, three are of particular note.

#### High Velocity Beneath the Central Craters

Located slightly south-east of the central craters, the high-velocity anomaly observed in Fig. 6 is the widely known as the HVB (e.g., Patanè et al. 2003, 2006, 2011; Martínez-Arevalo et al. 2005; Alparone et al. 2012). This anomaly extends up to 18 km depth below the summit region and southern part of the Valle del Bove and has appeared in all previous tomographic analyses. The proposed volume has differed between studies, averaging ~600 km^3^ up to 10 km of depth (e.g., Chiarabba et al. 2004; Patanè et al. 2006). Patanè et al. (2006) defined the shape of the HVB in detail and established a *V*_p_ ranging from 5.5 to 6.7 km/s between 2 and 7 km b.s.l. Our results are clearly in agreement with their observations. The origin of the anomaly is interpreted to be old magmatic intrusions, with the HVB representing the main structural feature beneath the volcano, reflecting its intense volcanic history and the accumulation of a very large volume of non-erupted volcanic material. Detailed studies of its composition have been performed; for example, Corsaro et al. (2014) analysed gabbroic xenoliths that may be originated from the HVB and inferred the slow solidification of a mafic magma.

#### High-Velocity South-east of Mt. Etna

For the first time, we have highlighted a high-velocity anomaly (5.5–6.5 km/s) located to the south-east of the Mt. Etna area (Fig. 6a). This high-velocity anomaly is oriented NE–SW (Fig. 6c) and is probably associated with the main structural features (NE–SW trending faults), suggesting interplay between submarine volcanic manifestations and tectonic setting. This body, extending from ~4.0–14 km b.s.l., confirms observations of a large and intense magnetic positive anomaly (>700 nT) related to deep sources (Cavallaro et al. 2016) and evidenced by the magnetic survey carried out during the TOMO-MT. ETNA experiment. This evidence is compatible with a plumbing system feeding the ancient shield volcano located offshore of the Timpe area (Corsaro et al. 2002; Chiocci et al. 2011), which has undergone significant erosive and tectonic processes since its formation >220 ka ago (Branca et al. 2011). It is the availability of these complementary data sources that have allowed our interpretation of this anomaly, despite its location near the edge of our resolvable area.

By the analysis of multi-channel seismic (MCS) reflection profiles performed during the TOMO-MT. ETNA experiment, we investigated the possible relationship between this high-velocity anomaly and an upwelling of the Moho, as suggested by Nicolich et al. (2000) for the Ionian margin of Sicily at up to 17 km b.s.l. Moreover, we also analysed the probable connection between the HVB and this high-velocity anomaly at ~8–10 km depth (Fig. 6c).

#### Low-Velocity West of the Central Craters

The low-velocity anomaly (Fig. 6) located west of the central craters, which was previously observed by Aloisi et al. (2002) and correlated with the anomalous *b* volume revealed by Murru et al. (1999) and normal-low *V*_p_ and low *Q*_p_ identified by De Gori et al. (2005) can be interpreted as a magmatic fluids storage area. It is interesting to notice that in the surroundings this low-velocity anomaly, recently (August 2017) several seismic swarms have been taking place. At the present, the real origin of these series is under deep analysis, and potential magmatic origin is not yet excluded.

## Conclusions

A reconstruction of the crustal structure of the north-eastern Sicily, including the volcanic environments of Mt. Etna and the Aeolian Islands, is proposed based on tomographic results from data acquired during the 2014 TOMO-MT. ETNA experiment. The results presented here correspond to a joint active passive tomographic inversion of 184,797 active P-phase and 11,802 passive P-phase first arrivals, recorded over a very short period of time. This snapshot has allowed us to discriminate individual volcanic structures without averaging them through time; however, we propose that further joint analyses, including an enlarged passive dataset, would not significantly change with time.

In addition to clearly delineating the HVB (Anomaly 5), which has a distinctive feature of several tomography studies at Mt. Etna, other significant features imaged by this study included a high-velocity anomaly-oriented NE–SW and located under the south-eastern sector of the volcano (Anomaly 6) and a low-velocity zone to the west of the central craters (Anomaly 8). Anomaly 6 overlaps an intense magnetic positive anomaly and could represent the plumbing system of an extinct shield volcano located offshore of Mt. Etna or, alternatively, could be related to an upwelling of the Moho. In contrast, Anomaly 8 likely represents a volume of magmatic fluid stored within the crust.

There results also detailed characteristics of a NW–SE crustal tear fault system (anomalies 2 and 3) between the Tyrrhenian and Ionian seas, which has formed owing to the motion of the western boundary of the Ionian slab as it slips below the Calabrian Arc. Finally, a low anomaly zone (Anomaly 4) beneath Panarea and Stromboli likely indicates magma accumulation beneath the Tyrrhenian volcanoes.

Our tomography has highlighted anomalies undiscovered in previous studies. In particular, a low-velocity ‘ring’ around Mt. Etna is clearly visible in shallower layers remains under investigation. Interpreting this feature requires further advanced analyses.

## Figures and Tables

**Fig. 7:**
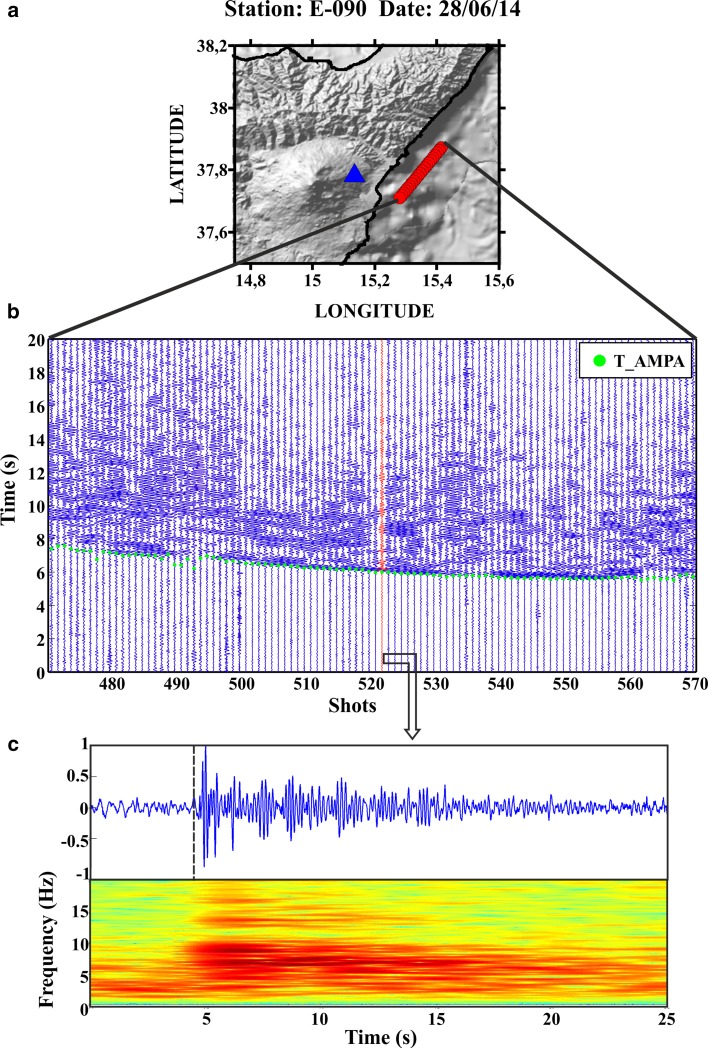
Set of active seismic signals recorded at a single station on Mt. Etna. Plot compares the signals of 100 shots and depicts picking homogeneity using the modified Adaptive Multiband Picking Algorithm (AMPA)

**Fig. 8:**
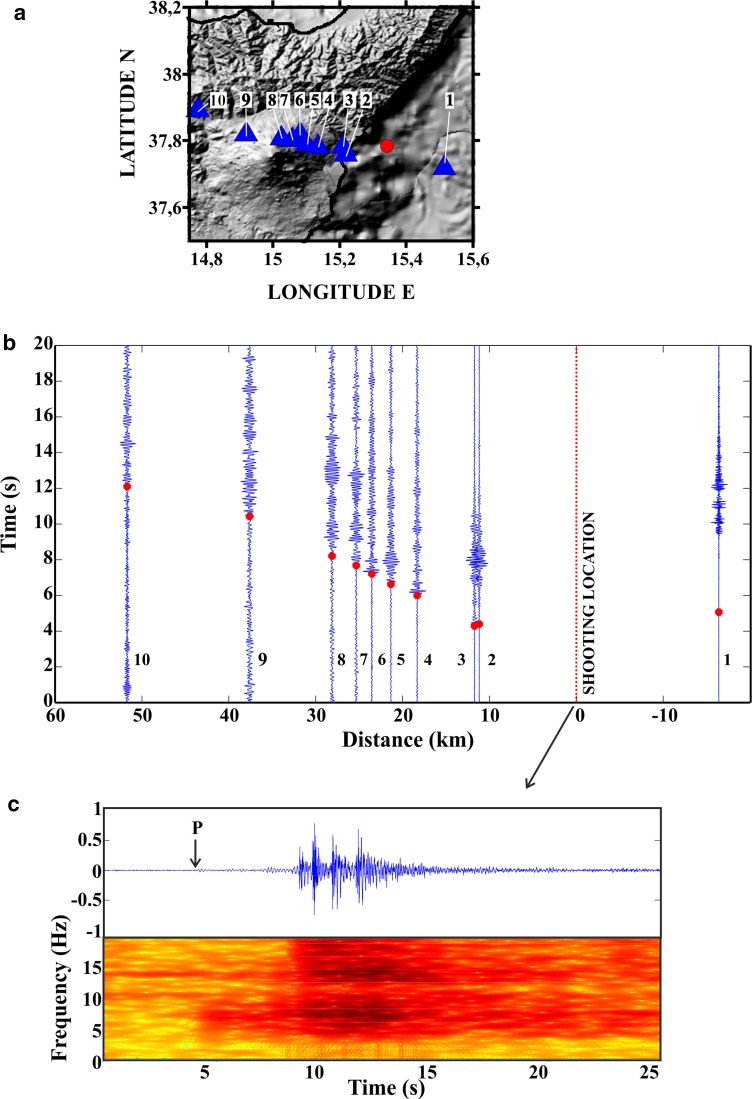
Example of data quality for active source seismic signals (air gun shots) compared to that of a single shot performed in front of Mt. Etna at a position aligned with the seismic stations. In this example, records are plotted starting from an ocean bottom seismometer (OBS) station (signal to the left of the shooting location, red line in **b**). The first P-wave onset can be recognized at distances of up to 50 km from the shoot and crossing the complex structure of Mt. Etna. The distance axis is reversed in order to align data with the orientation of panel ‘**a**’. **c** Seismogram and spectrogram of the record from the OBS seismometer, where the real P-wave onset does not correspond to the most energetic arrival but occurs a few seconds before. The observed high-energy signals are associated with arrival from the water layer and could produce a clear bias in the final image if mistakenly used as first onset

**Fig. 9:**
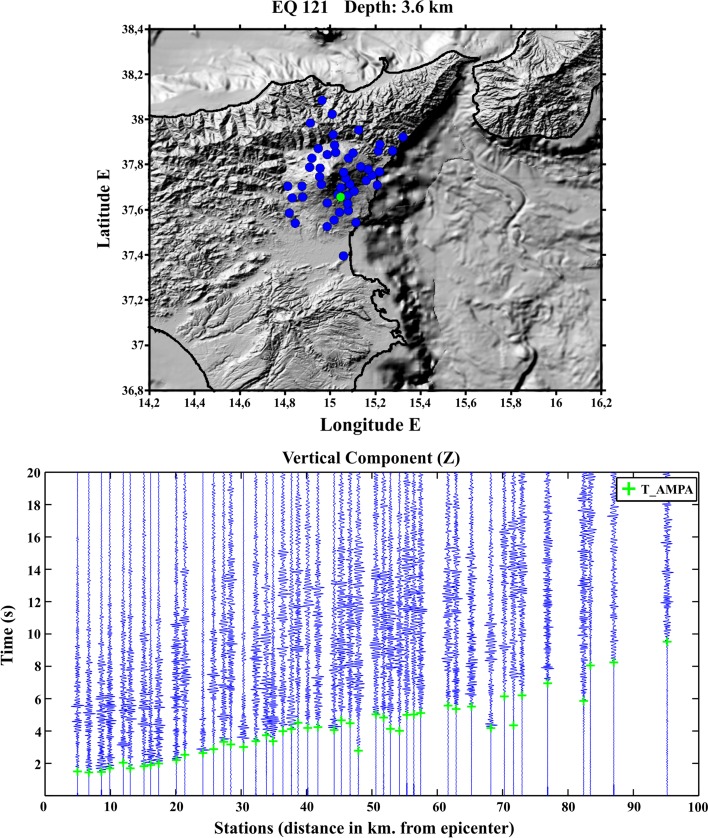
Examples of data quality for a passive-source seismic signal (a magnitude 2.0 [Ml] volcano tectonic [VT] earthquake registered on 12 July 2014 and located 3.6 km b.s.l. beneath Mt. Etna) recorded at different on-land seismic stations. The Adaptive Multiband Picking Algorithm (AMPA) software was able to identify the first P-wave onset at distances of almost 100 km from the source. Some stations placed at similar distances (e.g., around 50 km distance) but with different azimuths show differences among the first P-wave onsets. These differences reflect strong regional velocity contrasts

**Fig. 10:**
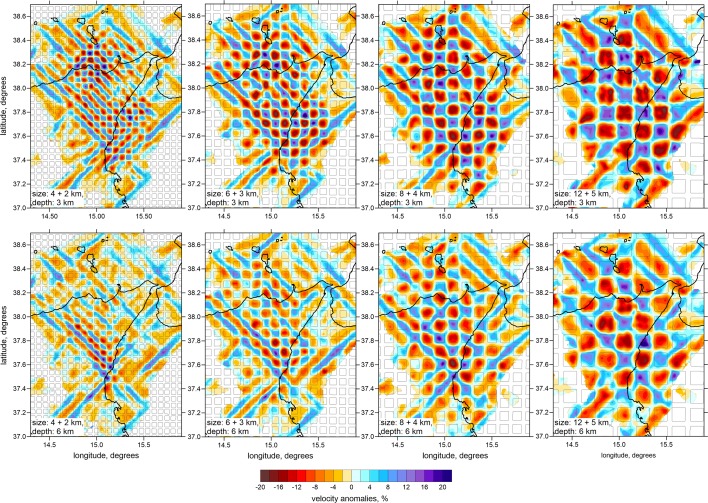
Checkerboard tests with different sizes of anomalies for checking the horizontal resolution in the cases of joint active and passive data inversions. In all cases, the anomalies remain unchanged with depth. The recovered results are shown at 3- and 6-km-depth b.s.l. The horizontal sizes of anomalies and the widths of the empty space between them are indicated in figure captions by two numbers. The original anomalies are indicated with grey lines

**Fig. 11:**
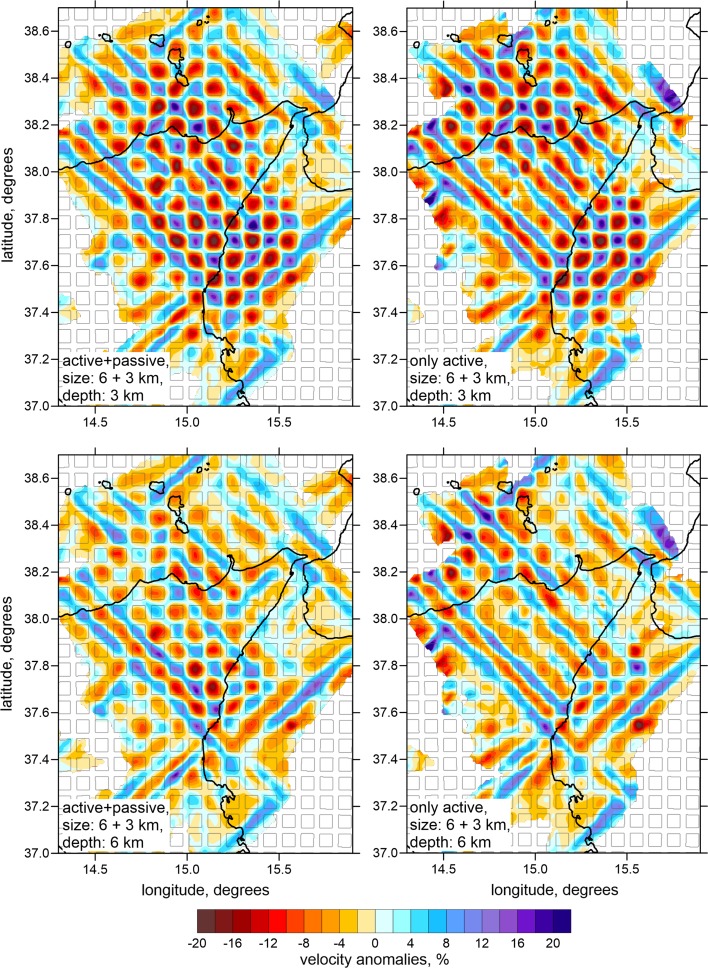
Comparison of the checkerboard tests in the cases of using both active and passive data (left column, same model as in second column in Fig. 10) and only active data (right column). In all cases, the anomalies remain unchanged with depth. The recovered results are shown at 3- and 6-km-depth b.s.l. The original anomalies are indicated with grey lines. It can be seen that adding the passive data considerably improve the resolution in the onshore parts, especially in deeper sections

**Fig. 12:**
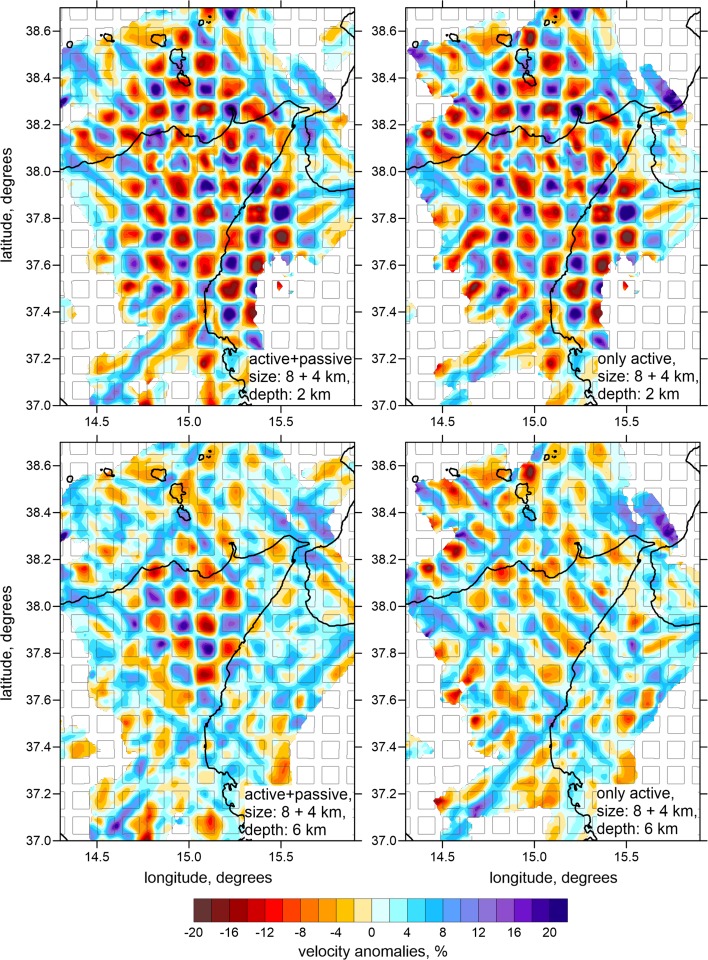
Checkerboard test for checking the vertical resolution in two cases: joint inversion of active and passive data (left column) and the inversion of solely active data (right). In both these cases, the anomalies change the sign at 4 km depth. The locations of the original anomalies are indicated with grey lines. It can be seen that for the shallow section, the resolution is determined mostly by active data and identical in both cases, whereas for the deeper section, the satisfactory resolution is only in area where the passive data are available

**Fig. 13:**
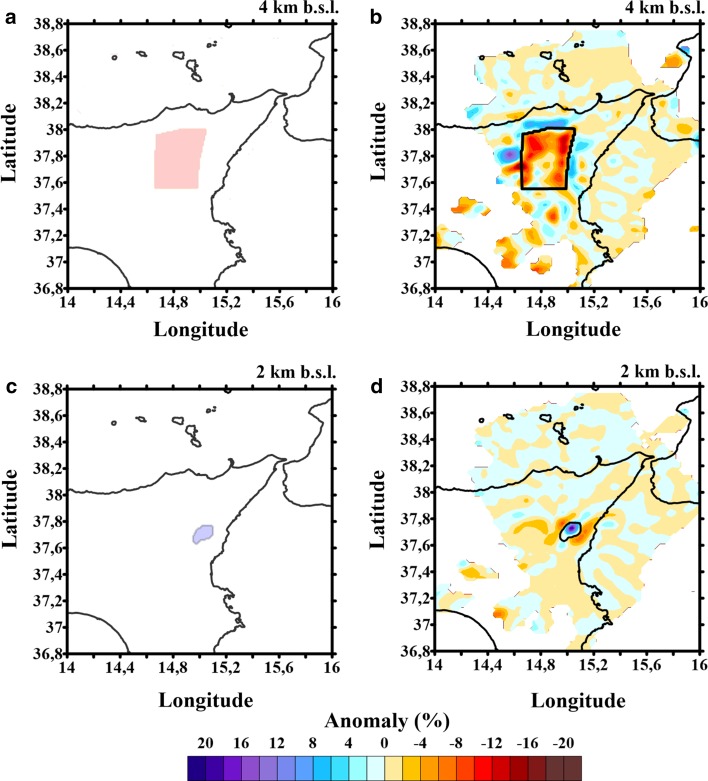
Images of the free anomaly test performed for Region 1 (whole study area), where two bodies with different velocity contrasts were numerically modelled to check the capability of our data to resolve them. Returning results are very close to the starting models for both high and low velocities

**Fig. 14:**
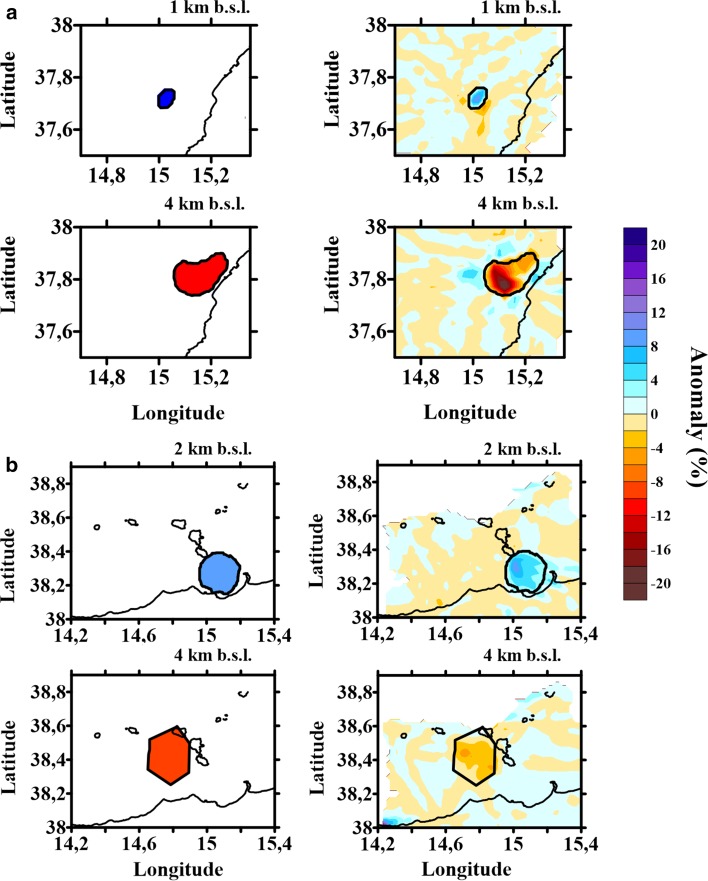
Images of the free anomaly test performed for **a** Region 2 (Aeolian Volcanic archipelago) and **b** Region 3 (Mt. Etna and surrounding areas), where for each, two bodies with different velocity contrasts were numerically modelled to check the capability of our data to resolve them. Returning results are very close to the starting models for both high and low velocities

**Fig. 15:**
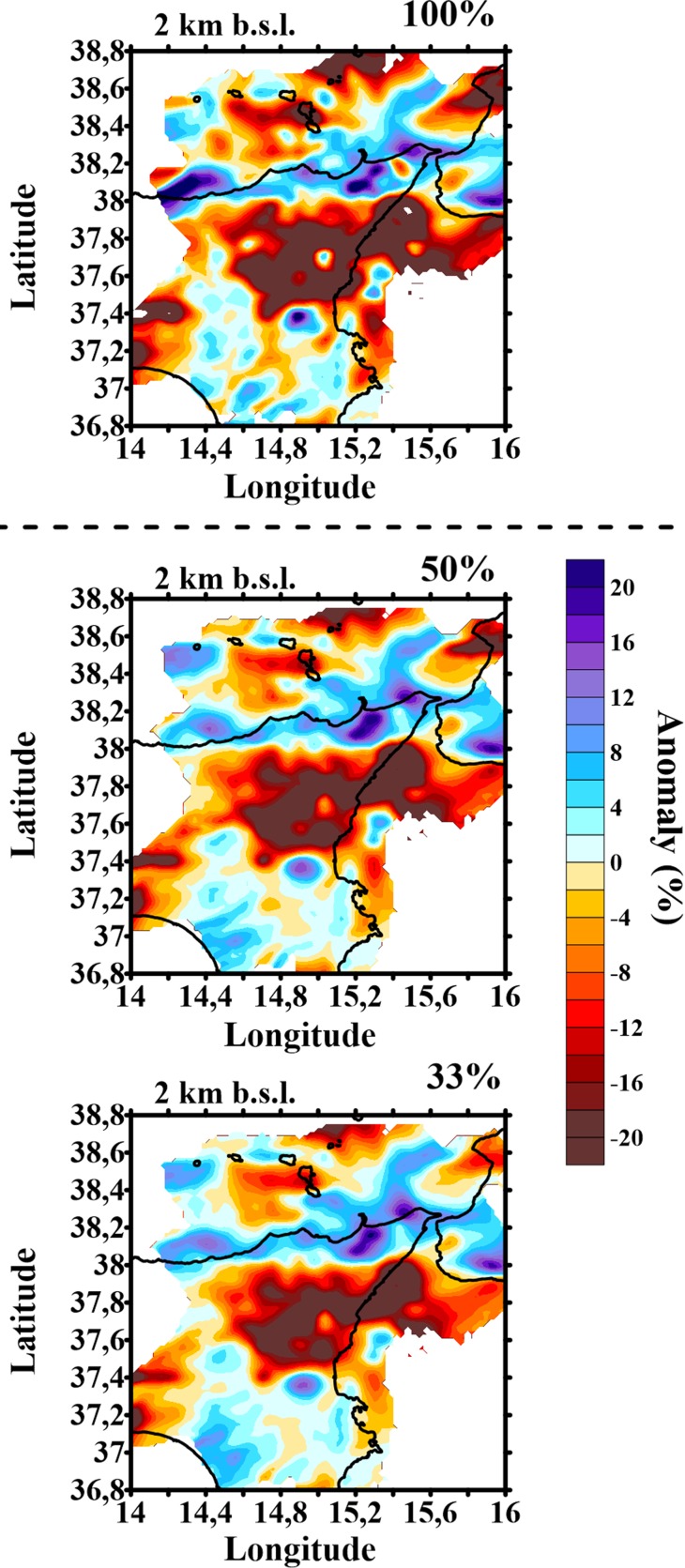
Jackknifing test for Region 1 (whole study area). Upper figure shows the final image using 100% of the available data. Lower figures show final images using random subsets created by eliminating 50 and 67% of the data. Even with large reductions in data, the final images remained stable, indicating the quality of our data and inversion

**Fig. 16:**
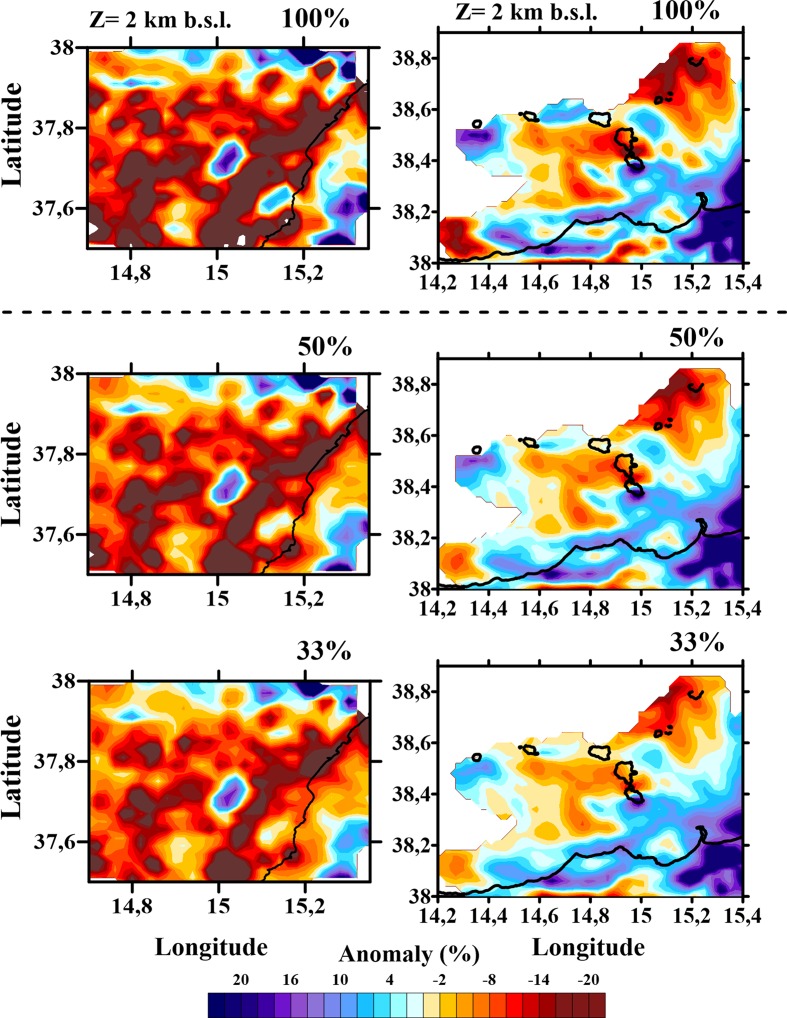
Jackknifing test for Region 2 (Aeolian Volcanic archipelago; right column) and Region 3 (Mt. Etna and surrounding area; left column). Upper figures show the final image using 100% of the available data. Lower figures show final images using random subsets created by eliminating 50 and 67% of the data. Even with large reductions in data, the final images remained stable, indicating the quality of our data and inversion

**Fig. 17:**
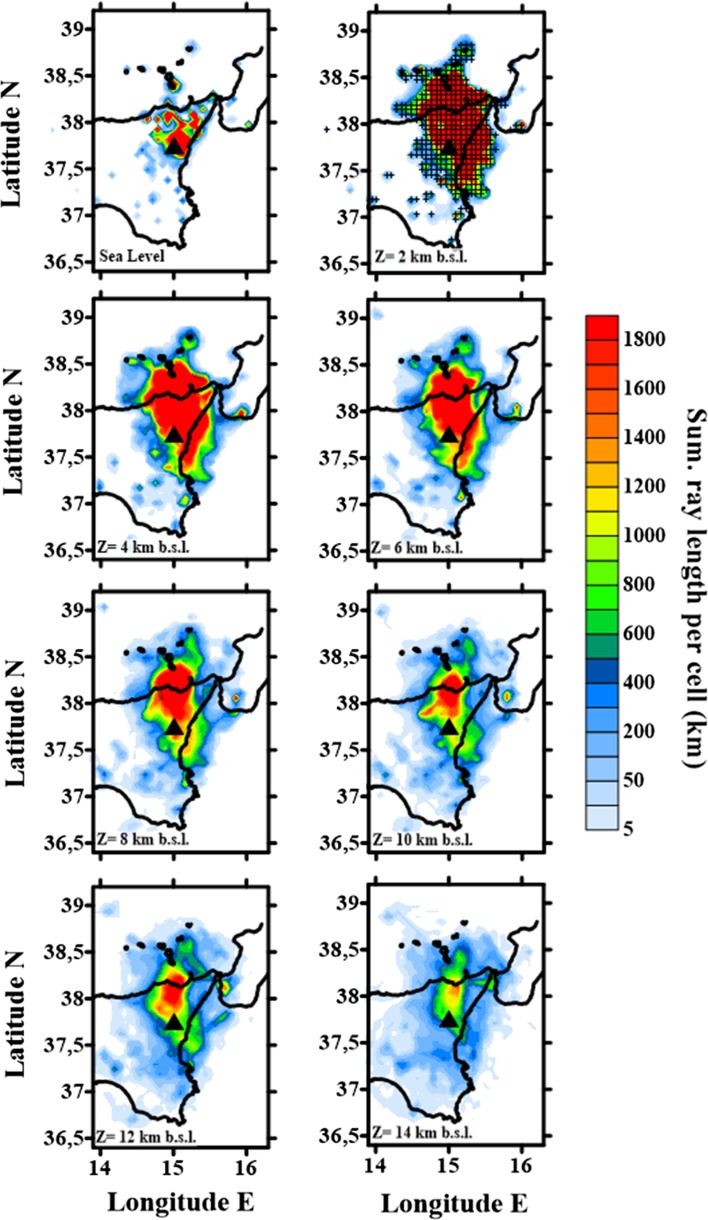
Colour scale surface projection of ray density for Region 1 (whole study region). The highest density grids are crossed by >1600 seismic rays. Lower-density grids are crossed by >50 seismic rays. White areas are those with >less than 5 rays (or no rays)

**Fig. 18:**
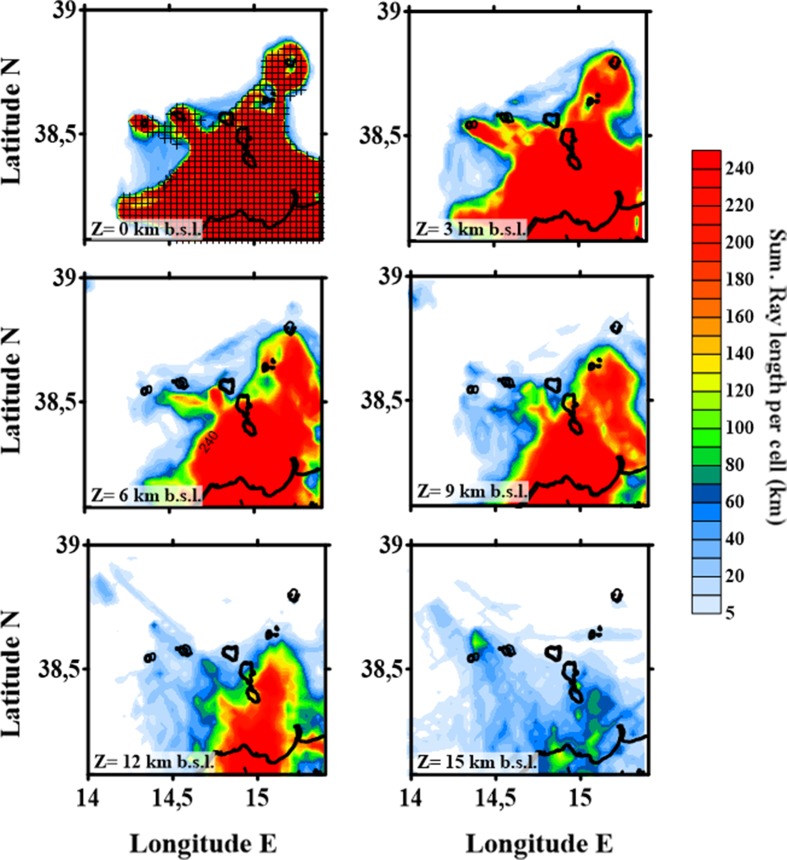
Colour scale surface projection of ray density for Region 2 (Aeolian Volcanic archipelago). Grid size is lower than that of Region 1 (Fig. 17). The highest density grids are crossed by >200 seismic rays. Lower-density grids are crossed by >20 seismic rays. White areas are those with >less than 5 rays (or no rays)

**Fig. 19:**
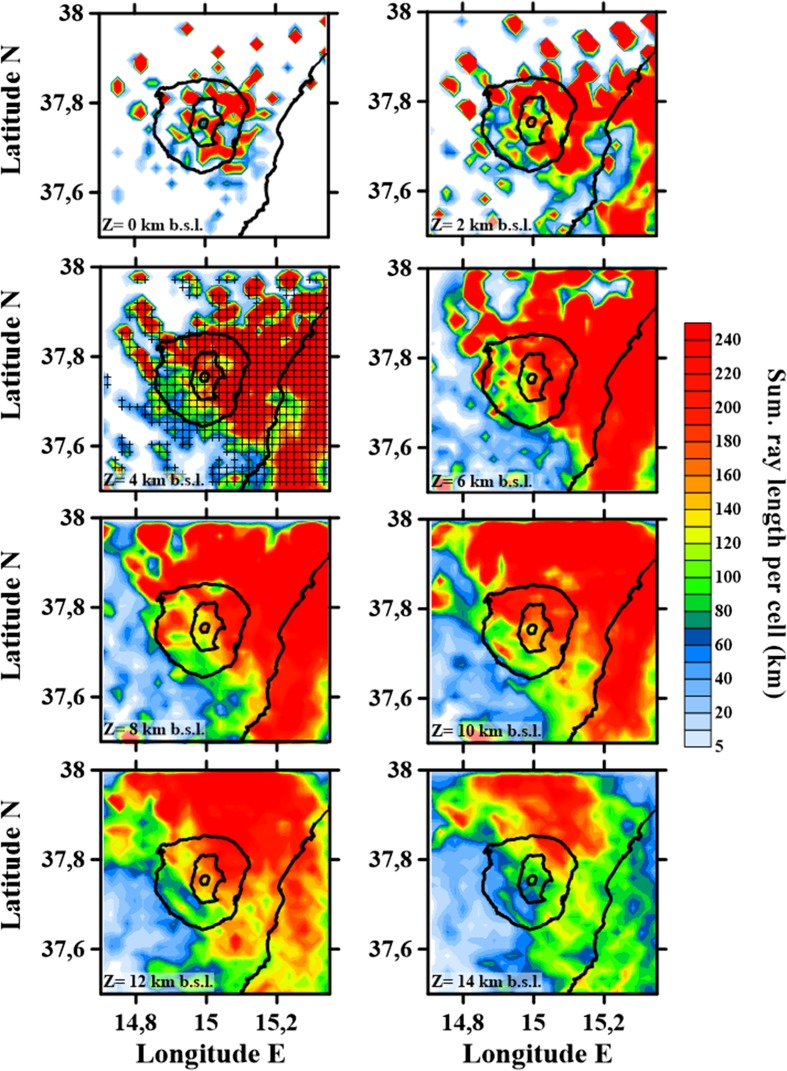
Colour scale surface projection of ray density for Region 3 (Mt. Etna and surrounding area). Grid size is lower than that of regions 1 (Fig. 17) and 2 (Fig. 18). The highest density grids are crossed by >250 seismic rays. White areas are those with >less than 5 rays (or no rays)

## Appendix 1: Data Quality

In Fig. 7, we plot 100 active shots recorded at a single station placed near Mt. Etna volcano. This figure allows us to observe the stability of the automatic procedure for providing coherent P-wave arrivals.

In Fig. 8, we plot a single active shot recorded along a profile of seismic stations that cross the volcanic structure of Mt. Etna, starting with an ocean bottom seismometer (OBS) station. This figure illustrates the mean maximum distance that an active source can be recorded with high quality, and which of the P-wave onset picking procedures worked correctly, in this case up to 55 km.

In Fig. 9, we plot seismograms from a single VT earthquake at Mt. Etna. This event had a magnitude of 2.0 (Ml) and was recorded by several seismic stations. In this figure, we illustrate how the Adaptive Multiband Picking Algorithm (AMPA) is able to detect P-wave first onset up to 100 km from the source. It is important to note that stations at similar distances (e.g., ~50 km distance) but different azimuths show differences among the first P-wave onsets. These differences reflect strong velocity contrasts in the region and confirm the strong lateral velocity contrasts observed in our models.

## Appendix 2: Configuration Parameters

We provide the different parameters used to run the inversions. Table 1 shows the numbers obtained for the different tests.

Dataset:

1. Active
2. Passive
3. Joint

REFMOD (1-D velocity model):

1. Fast_1
2. Medium_1
3. Slow_1
4. Fast_2
5. Medium_2
6. Slow_2
7. Fast_3
8. Medium_3

Smoothing:

1. 1.0
2. 0.5
3. 1.5
4. 0.8

Weights for active/passive:

1. 1.0/1.0
2. 0.75/1.0
3. 0.5/1.0
4. 0.25/1.0
5. 0.35/1.0

Tests:

1. DATASET 3 Complete
2. DATASET 3 EQ < 40 km
3. DATASET 3 al 50% (Jackknifing test)

## Appendix 3: Synthetic tests

Here, we present a complete framework of synthetic tests used to assess the TOMO-ETNA dataset and resulting tomographic images.

### Checkerboards

To determine optimal spatial resolution and smearing effects, we performed a series of checkerboard tests. In Fig. 10, there are checkerboard anomalies, which remain unchanged with depth and have different lateral sizes. This test helps us to estimate the min size of anomalies in different parts of the study area. In Fig. 11, we consider the cases with and without adding the passive data. It can be seen that adding the passive data considerably improves the resolution in onshore areas, especially at great depths. In order to enlighten vertical resolution, we plot Fig. 12, where the anomalies change the sign at 4 km b.s.l. Here, we also consider the cases with and without the passive data. For the shallow section, adding the passive data does not change the result, whereas for the deeper section, only active data are not sufficient to achieve sufficient resolution.

### Free Anomaly Test

To determine the reliability of resulting anomalies, we inverted two synthetic anomalies, one of them similar in size and shape to those expected from the real inversions, in order to check the capacity of the model (Fig. 13b, d). Positive and negative free anomalies were set for the larger area (Fig. 13). These anomalies were designed to be larger than the grid spacing in order to avoid 1-node anomalies.

A similar configuration was inverted for the smaller region around Mt. Etna (Fig. 14 left column). Again, the sizes of the synthetic anomalies took into account the grid spacing in order to reproduce realistic anomalies. The results of this test confirm the high quality of our inversions. For the Aeolian Islands, positive and negative synthetic anomalies with geometries similar to the real anomalies were convincingly resolved (Fig. 14b left column), again confirming the robustness and reliability of the results.

### Jackknifing Test

Noise detection and reduction is important for tomographic analysis. Noise reduction is performed during the preprocessing and picking of data. Determining the contribution of remnant noise to the final results requires a jackknifing test. This synthetic approach is based on inverting random subsets of data and comparing the final results. Noise disturbance dramatically modifies the results from different subsets, while stable results with low noise should not be modified when reducing the inverted dataset. Some studies (e.g., García-Yeguas et al. 2014) take random subsets from the odd or even travel times of the complete dataset. For the TOMO-ETNA dataset, we performed this test using 50 or 33% of the travel times and compared the full dataset inversion for a single layer at 2 km b.s.l. (Fig. 15). The results confirm low noise disturbance in the final inversion, ensuring confidence in the quality and reliability of our tomographic results. Furthermore, jackknifing tests for the Mt. Etna region (Fig. 16 left column) and for the Aeolian Islands (Fig. 16 right column) show no significant changes when compared to the complete dataset inversion, thus confirming the stability of the resulting model.

### Ray Coverage

The ray density of the final solution gives a clear view of model resolution as it shows ray coverage for the whole area; therefore, areas with a higher ray density have more final results. The high density of ray distribution in each of the different regions ensured the quality of the input dataset (Figs. 17, 18, 19). In contrast, many other seismic studies have declared good coverage with less than 10 rays. Figure 17 shows the ray density for Region 1 separated into different horizontal layers. These results are in agreement with the other synthetic tests, confirming that the well-resolved area is essentially the same in both checkerboard and ray density maps. Ray density maps for regions 2 and 3 are illustrated in Figs. 18 and 19, respectively.
